# From cycloheptathiophene-3-carboxamide to oxazinone-based derivatives as allosteric HIV-1 ribonuclease H inhibitors

**DOI:** 10.1080/14756366.2018.1523901

**Published:** 2018-10-26

**Authors:** Serena Massari, Angela Corona, Simona Distinto, Jenny Desantis, Alessia Caredda, Stefano Sabatini, Giuseppe Manfroni, Tommaso Felicetti, Violetta Cecchetti, Christophe Pannecouque, Elias Maccioni, Enzo Tramontano, Oriana Tabarrini

**Affiliations:** a Department of Pharmaceutical Sciences, University of Perugia, Perugia, Italy;; b Department of Life and Environmental Sciences, University of Cagliari, Cittadella Universitaria di Monserrato, Monserrato, Cagliari, Italy;; c Department of Chemistry, Biology and Biotechnology, University of Perugia, Perugia, Italy;; d Rega Institute for Medical Research, Laboratory of Virology and Chemotherapy, K.U. Leuven, K.U. Leuven, Leuven, Belgium;; e Istituto di Ricerca Genetica e Biomedica, Consiglio Nazionale delle Ricerche (CNR), Monserrato, Italy

**Keywords:** Allosteric inhibitors, antiviral agents, thieno[2,3-*d*][1,3]oxazin-4-one derivatives, HIV-1 ribonuclease H

## Abstract

The paper focussed on a step-by-step structural modification of a cycloheptathiophene-3-carboxamide derivative recently identified by us as reverse transcriptase (RT)-associated ribonuclease H (RNase H) inhibitor. In particular, its conversion to a 2-aryl-cycloheptathienoozaxinone derivative and the successive thorough exploration of both 2-aromatic and cycloheptathieno moieties led to identify oxazinone-based compounds as new anti-RNase H chemotypes. The presence of the catechol moiety at the C-2 position of the scaffold emerged as critical to achieve potent anti-RNase H activity, which also encompassed anti-RNA dependent DNA polymerase (RDDP) activity for the tricyclic derivatives. Benzothienooxazinone derivative **22** resulted the most potent dual inhibitor exhibiting IC_50_s of 0.53 and 2.90 μM against the RNase H and RDDP functions. Mutagenesis and docking studies suggested that compound **22** binds two allosteric pockets within the RT, one located between the RNase H active site and the primer grip region and the other close to the DNA polymerase catalytic centre.

## Graphical Abstract

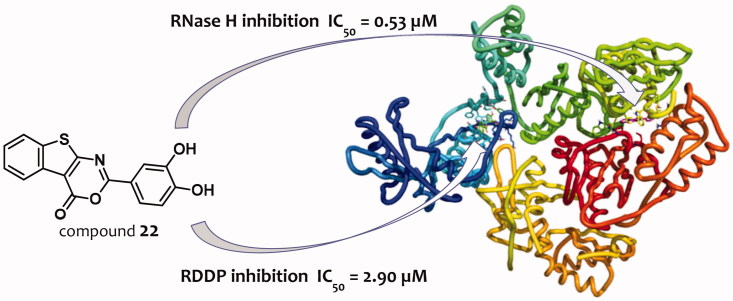

## Introduction

Despite many efforts in declining morbidity and mortality in AIDS patients through the combination antiretroviral therapy (cART), controlling HIV infection remains a global health priority. cART significantly suppresses viral load, thus preventing the progression to advanced AIDS and improving both the quality and expectancy of patients life^1^^,^^2^. However, the benefits of such cocktail of drugs are often compromised by severe side effects and the need for a life-long treatment. Most important, neither a cure nor eradication of HIV infection is possible yet^3^, due to the development of latent but replication-competent viral forms. The number of newly infected people per year is constant and there is an overall increase of detected drug-resistant variants transmitted among antiretroviral treatment–naïve patients^4^. The resistance can affect multiple classes of drugs^5^, dramatically impairing the outcome of cART. Thus, the discovery of new agents is crucial. One approach to minimise the development of drug resistance may rely on compounds endowed with alternative mechanisms of action or new binding sites on traditional targets to complement/enrich the current drugs cocktails^6^.

The reverse transcriptase (RT) represents the most exploited target within the anti-HIV therapy, with more than half of the approved drugs inhibiting this enzyme. RT is a heterodimeric (p66/p51) multifunctional enzyme responsible for the conversion of the viral single-stranded RNA genome into the double-stranded DNA. This activity is essentially accomplished by the RNA- and DNA-dependent DNA polymerase (RDDP and DDDP) and ribonuclease H (RNase H) functions, whose active sites reside on the p66 subunit. While the polymerase catalyses nucleotidyl transfer reactions for the DNA synthesis, the RNase H hydrolyses the RNA strand within the RNA:DNA hybrid generated during the reverse transcription. Both functions are necessary for successful reverse transcription, but while the polymerase function has been targeted by all the RT inhibitors in therapy, no drug inhibiting the RT-associated RNase H function has entered clinical development up to now, despite the fact that its abrogation^7^ and selective inhibition^8^^,^^9^ has been proved to compromise the viral infectivity. Since 1990 several RNase H inhibitors (RNHIs) have been identified that^10^^,^^11^, based on their mechanism of action, can be classified in active site and allosteric inhibitors. The wider class of RNHIs is represented by compounds acting through the chelation of the divalent cations present at the RNase H active site and required as cofactors in enzyme catalysis. Some of them were originally identified as inhibitors of the influenza virus endonuclease or HIV integrase (IN)^12^. Given the structural similarities between RNase H and IN active sites, they both can be targeted by some classes of compounds that act as metal coordinating agents^13–15^. Less populated is the class of allosteric RNHIs, which were identified through various approaches and include structurally different compounds, of which some endowed with dual activity against both RT-associated functions^16–19^. Targeting allosteric sites could be more advantageous to avoid the inhibition of related host enzymes.

**Figure 1.**
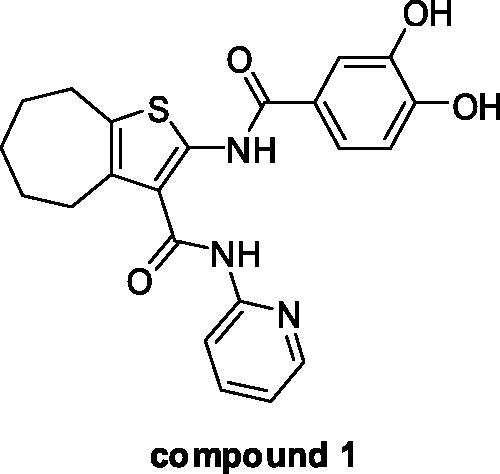
Chemical structure of cHTC compound **1**.

As a part of our work aimed at identifying innovative anti-HIV compounds^20–23^, we have recently contributed to the allosteric RNHIs class reporting a series of cycloheptathiophene-3-carboxamide (**cHTC**) derivatives^24^, which were identified through a repurposing approach. Indeed, they were born as influenza virus inhibitors with the ability to disrupt the PA-PB1 subunits interaction of the viral RNA polymerase^25–28^, and then, based on the strict structural similarity with a series of known RNHIs^29^, they were assayed for this activity. From the initial screening, compounds endowed with good inhibitory activity emerged whose structural optimization led to the catechol derivative **1** (Figure 1), which, showing an IC_50_ value of 0.84 µM, ranked from the most active allosteric RNHIs.

Many other cHTCs showed IC_50_ values in the low micromolar range, confirming the cycloheptathiophene ring particularly suitable to inhibit this function of the RT enzyme. They were unable to chelate the Mg^2+^ ions excluding any interaction with the RNase H catalytic centre. In addition, they did not inhibit RT-associated RDDP function with the only exception of compound **1**, which, however, showed a 20-fold weaker potency against the polymerase activity with respect to RNase H activity. Docking simulations performed to achieve insights on these selective inhibitors showed that they recognised a new allosteric binding site including residue Q500 that played a key role in the binding. Q500 was reported as responsible for the RNA:DNA hybrid binding thus compounds interacting with this residue or residues in its vicinity could potentially interfere with duplex accommodation in the RNase H active site^30^.

Having in hand good and selective RNase H allosteric inhibitors that act by recognising a new allosteric site, in this work we decided to further exploit the cHTC scaffold designing and synthesising a wide series of analogues.

## Materials and methods

### Chemistry

Starting materials, reagents, and solvents that were commercially available were used as supplied. The reactions were monitored by TLC on silica gel 60F254 (Merck) and visualised by UV and/or iodine. Flash chromatography columns were performed on Merck silica gel 60 (mesh 230–400). After extraction, organic solutions were dried by using anhydrous Na_2_SO_4_, filtered, and evaporator to dryness at reduced pressure with a Büchi rotary. Yields are of pure products and were not optimised. HRMS spectra were registered on Agilent Technologies 6540 UHD Accurate-Mass Q-TOF LC/MS, HPLC 1290 Infinity. Purities of target compounds were determined by UHPLC on Agilent Technologies 6540 UHD Accurate-Mass Q-TOF LC/MS, HPLC 1290 Infinity with DAD detector and evaluated to be ≥95%. HPLC conditions to assess the purity of final compounds were as follows: column, Phenomenex AERIS Widepore C4, 4.6 mm ×100 mm (6.6 μm); flow rate, 0.85 ml/min; acquisition time, 10 min; DAD 254 nm; oven temperature, 30 °C; gradient of acetonitrile in water containing 0.1% of formic acid (0 − 100% in 10 min). Nuclear magnetic resonance (NMR) spectra were recorded on Bruker Avance DRX-400 MHz using residual solvents such as dimethylsulphoxide (*δ* = 2.48) or chloroform (*δ* = 7.26) as an internal standard. Chemical shifts (*δ*) are reported in parts per million (ppm), and peak multiplicity are reported as s (singlet), d (doublet), t (triplet), q (quartet), p (pentet), hept (heptet), m (multiplet), or brs (broad singlet). Compounds **2**^25^, **3**^31^, and **5**^28^ were prepared as previously described.

### General procedure for demethylation (method A)

To a solution of the appropriate methoxy derivative (1.0 equiv) in dry CH_2_Cl_2_, a 1 M solution of BBr_3_ in CH_2_Cl_2_ (6.0 equiv) was added dropwise maintaining the temperature at 0 °C. The reaction mixture was stirred at room temperature (r.t.) until no starting material was detected by TLC, and then quenched with MeOH and water. The organic solvent was removed under vacuum affording a residue, which was filtered and purified as described below.

2-Amino-N-(2-hydroxyphenyl)-5,6,7,8-tetrahydro-4*H*-cyclohepta[*b*]thiophene-3-carboxamide (**4**). The title compound was prepared starting from **3**^31^ through Method A (16 h) and purified by crystallization from EtOH, in 65% yield as light-yellow crystals; ^1^H NMR (DMSO-*d*_6_, 400 MHz): *δ* 1.45–1.60 (m, 4H, cycloheptane CH_2_), 1.70–1.80 (m, 2H, cycloheptane CH_2_), 2.50–2.60 and 2.70–2.80 (m, each 2H, cycloheptane CH_2_), 6.20 (bs, 2H, NH_2_), 6.70 (t, *J* = 7.6 Hz, 1H, aromatic CH), 6.80–6.90 (m, 2H, aromatic CH), 7.90 (d, *J* = 7.9 Hz, 1H, aromatic CH), 8.60 (s, 1H, NH), 9.90 (s, 1H, OH); 13 C NMR (DMSO-*d*_6_, 101 MHz): *δ* 27.3, 28.0, 28.5, 28.7, 32.0, 113.6, 115.5, 119.5, 121.0, 121.7, 124.0, 127.4, 136.3, 147.2, 154.6, 164.1; HRMS: calcd for C_16_H_18_N_2_O_2_S 303.1168 (M + H)^+^, found 303.1169.

### General procedure for carbodiimide formation (method B)

A solution of the appropriate synthone (1.0 equiv) in dry pyridine was added to the suitable benzoyl chloride (2.0 equiv). The reaction mixture was maintained at r.t. until no starting material was detected by TLC. After cooling, the reaction mixture was poured into ice/water, obtaining a precipitate which was filtered and purified as described below.

2-[(4-Chlorobenzoyl)amino]-5,6,7,8-tetrahydro-4*H*-cyclohepta[*b*]thiophene-3-carboxamide (**9**). The title compound was prepared starting from **31**^32^ through Method B (16 h) using 4-chlorobenzoyl chloride, and purified by flash chromatography eluting with cyclohexane/EtOAc (7:3), in 35% yield as light-yellow solid; ^1^H NMR (DMSO-*d*_6_, 400 MHz): *δ* 1.50–1.60 (m, 4H, cycloheptane CH_2_), 1.70–1.80 (m, 2H, cycloheptane CH_2_), 2.65–2.70 and 2.75–2.80 (m, each 2H, cycloheptane CH_2_), 7.50 (bs, 2H, NH_2_), 7.60 (d, *J =* 8.5 Hz, 2H, aromatic CH), 7.80 (d, *J* = 8.5 Hz, 2H, aromatic CH), 11.75 (s, 1H, NH); ^13 ^C NMR (DMSO-*d*_6_, 101 MHz): δ 27.5, 27.9, 28.4, 28.6, 31.9, 122.2, 129.4, 129.4, 131.5, 131.8, 135.7, 137.4, 137.4, 162.1, 167.8; HRMS: calcd for C_17_H_17_ClN_2_O_2_S 349.0778 (M + H)^+^, found 349.0774.

2-[(3,4-Dimethoxybenzoyl)amino]-5,6,7,8-tetrahydro-4*H*-cyclohepta[*b*]thiophene-3-carboxamide (**32**). The title compound was prepared starting from **31**^32^ through Method B (4 h) using 3,4-methoxybenzoyl chloride, and purified by treatment with Et_2_O, in 32% yield as white solid. 1H NMR (DMSO-*d*_6_, 400 MHz): δ 1.50–1.60 (m, 4H, cycloheptane CH_2_), 1.70–1.80 (m, 2H, cycloheptane CH_2_), 2.60–2.70 and 2.75–2.85 (m, each 2H, cycloheptane CH_2_), 3.80 (s, 6H, OCH_3_), 7.00–7.10 (m, 1H, aromatic CH),7.30–7.60 (m, 3H, aromatic CH and NH_2_), 11.75 (s, 1H, NH).

2-[(2-Methoxybenzoyl)amino]-5,6,7,8-tetrahydro-4*H*-cyclohepta[*b*]thiophene-3-carboxamide (**7**). The title compound was prepared starting from **31**^32^ through Method B (16 h) using 2-methoxybenzoyl chloride, and purified by flash chromatography eluting with cyclohexane/EtOAc (5:5), in 57% yield as white solid; ^1^H NMR (DMSO-*d*_6_, 400 MHz): δ 1.50–1.60 (m, 4H, cycloheptane CH_2_), 1.65–1.85 (m, 2H, cycloheptane CH_2_), 2.60–2.70 and 2.75–2.85 (m, each 2H, cycloheptane CH_2_), 4.00 (s, 3H, OCH_3_), 7.00–7.10 (m, 1H, aromatic CH), 7.20 (d, *J =* 7.6 Hz, 1H, aromatic CH), 7.30–7.45 (m, 1H, aromatic CH), 7.60 (bs, 2H, NH_2_), 8.05 (d, *J =* 6.3 Hz, 1H, aromatic CH), 12.10 (s, 1H, NH); ^13 ^C NMR (DMSO-*d*_6_, 101 MHz): δ 27.6, 28.0, 28.5, 28.6, 32.1, 56.4, 112.8, 119.4, 121.4, 121.6, 130.8, 131.8, 134.4, 135.2, 137.0, 157.8, 160.8, 167.7; HRMS: *m/z* calcd for C_18_H_20_N_2_O_3_S 345.1274 (M + H)^+^, found 345.1269.

2-[(3,4-Dihydroxybenzoyl)amino]-5,6,7,8-tetrahydro-4*H*-cyclohepta[*b*]thiophene-3-carboxamide (**6**). The title compound was prepared starting from **32** through Method A (4 h) and purified by flash chromatography eluting with CHCl_3_/MeOH (95:5), in 49% yield as light-yellow solid; ^1^H NMR (DMSO-*d*_6_, 400 MHz_,_): *δ* 1.50–1.65 (m, 4H, cycloheptane CH_2_), 1.70–1.85 (m, 2H, cycloheptane CH_2_), 2.60–2.70 and 2.75–2.85 (m, each 2H, cycloheptane CH_2_), 6.85 (d, *J* = 8.1 Hz, 1H, aromatic CH), 7.15 (d, *J* = 8.1 Hz, 1H, aromatic CH), 7.25–7.30 (m, 1H, aromatic CH), 7.40 (bs, 2H, NH_2_), 9.40 and 9.60 (s, each 1H, OH), 11.70 (s, 1H, NH); ^13 ^C NMR (DMSO-*d*_6_, 101 MHz): *δ* 27.5, 27.9, 28.6, 31.9, 114.8, 115.9, 119.4, 120.2, 123.8, 130.5, 135.2, 139.4, 145.9, 150.1, 162.7, 168.4; HRMS: *m/z* calcd for C_17_H_18_N_2_O_4_S 347.1066 (M + H)^+^, found 347.1061.

2-[(2-Hydroxybenzoyl)amino]-5,6,7,8-tetrahydro-4*H*-cyclohepta[*b*]thiophene-3-carboxamide (**8**). The title compound was prepared starting from **7** through Method A (4 h) and purified by flash chromatography eluting with cyclohexane/EtOAc (6:4), in 74% yield as white solid; ^1^H NMR (DMSO-*d*_6_, 400 MHz,): *δ* 1.50–1.65 (m, 4H, cycloheptane CH_2_), 1.70–1.85 (m, 2H, cycloheptane CH_2_), 2.60–2.70 and 2.70–2.80 (m, each 2H, cycloheptane CH_2_), 6.90–7.00 (m, 2H, aromatic CH), 7.30–7.50 (m, 3H, aromatic CH and NH_2_), 7.90 (dd, J = 1.6 and 7.8 Hz, 1H, aromatic CH), 11.75 (s, 1H, OH), 12.10 (s, 1H, NH); ^13 ^C NMR (DMSO-*d_6_*, 101 MHz): δ 27.6, 28.2, 28.6, 28.7, 32.2, 117.1, 117.7, 120.0, 122.0, 130.7, 131.3, 134.1, 135.1, 136.5, 156.7, 161.8, 167.5; HRMS: *m/z* calcd for C_17_H_18_N_2_O_3_S 331.1117 (M + H)^+^, found 331.1146.

Ethyl 2-[(3-methoxybenzoyl)amino]-5,6,7,8-tetrahydro-4*H*-cyclohepta[*b*]thiophene-3-carboxylate (**40**). The title compound was prepared starting from **33**^33^ by Method B (4 h) and purified by treatment with cyclohexane, in 44% yield; ^1^H-NMR (CDCl_3_, 400 MHz) *δ* 1.40 (t, *J* = 7.1 Hz, 3H, CH_2_*CH_3_*), 1.60–1.70 (m, 4H, cycloheptane CH_2_), 1.75–1.85, 2.65–2.75, and 3.00–3.10 (m, each 2H, cycloheptane CH_2_), 3.80 (s, 3H, OCH_3_), 4.40 (q, *J* = 7.0 Hz, 2H, *CH_2_*CH_3_), 7.70–7.80 (m, 3H, aromatic CH), 12.15 (s, 1H, NH).

Ethyl 2-[(3,4-dimethoxybenzoyl)amino]-5,6,7,8-tetrahydro-4*H*-cyclohepta[*b*]thiophene-3-carboxylate (**42**). The title compound was prepared starting from **33**^33^ by Method B (1 h) and purified by treatment with a Et_2_O/cyclohexane (1:2) mixture, in 100% yield; ^1^H NMR (DMSO-*d*_6_, 400 MHz) *δ* 1.25 (t, *J* = 7.0 Hz, 3H, CH_2_*CH_3_*), 1.50–1.60 (m, 4H, cycloheptane CH_2_), 1.75–1.80, 2.20–2.25, and 3.00–3.05 (m, each 2H, cycloheptane CH_2_), 3.80 (s, 6H, OCH_3_), 4.30 (q, *J* = 7.0 Hz, 2H, *CH_2_*CH_3_), 7.10–7.15 (m, 1H, aromatic CH), 7.45–7.50 (m, 2H, aromatic CH), 11.75 (s, 1H, NH).

Ethyl 2-[(3,4-dimethoxybenzoyl)amino]-4,5,6,7-tetrahydro-1-benzothiophene-3-carboxylate (**43**). The title compound was prepared starting from **34**^34^ by Method B (1 h) and purified by treatment with a Et_2_O/cyclohexane (1:2) mixture, in 96% yield; ^1^H NMR (DMSO-*d*_6_, 400 MHz) δ 1.25 (t, *J* = 7.0 Hz, 3H, CH_2_CH_3_), 1.60–1.70 (m, 4H, cyclohexane CH_2_), 2.55–2.60 and 2.65–2.70 (m, each 2H, cyclohexane CH_2_), 3.75 (s, 6H, OCH_3_), 4.25 (q, *J* = 7.0 Hz, 2H, CH_2_CH_3_), 7.05–7.10 (m, 1H, aromatic CH), 7.40–7.45 (m, 2H, aromatic CH), 12.00 (s, 1H, NH).

Ethyl 2-[(3,4-dimethoxybenzoyl)amino]-5,6-dihydro-4*H*-cyclopenta[*b*]thiophene-3-carboxylate (**44**). The title compound was prepared starting from **35**^34^ by Method B (1 h) and purified by treatment with a Et_2_O/cyclohexane (1:2) mixture, in 71% yield; ^1^H NMR (CDCl_3_, 400 MHz) δ 1.25 (t, *J* = 7.0 Hz, 3H, CH_2_*CH_3_*), 2.30–2.40 (m, 2H, cyclopentane CH_2_), 2.80–2.90 (m, 4H, cyclopentane CH_2_), 3.90 and 3.95 (s, each 3H, OCH_3_), 4.30 (q, *J* = 7.0 Hz, 2H, *CH_2_*CH_3_), 6.90 (d, *J* = 8.3 Hz, 1H, aromatic CH), 7.50 (dd, *J* = 2.2 and 8.3 Hz, 1H, aromatic CH), 7.65 (d, *J* = 2.2 Hz, 1H, aromatic CH), 12.00 (s, 1H, NH).

### General procedure for hydrolysis (method C)

A suspension of the appropriate ethyl thiophene-3-carboxylate (1.0 equiv) and LiOH (4.0 equiv) in a mixture H_2_O/THF (1:1) was maintained at 50 °C until no starting material was detected by TLC. After cooling the reaction mixture was acidified (pH 4–5) with 2 N HCl and the precipitate was filtered and purified as described below.

2-(Benzoylamino)-5,6,7,8-tetrahydro-4*H*-cyclohepta[*b*]thiophene-3-carboxylic acid (**47**). The title compound was prepared starting from **38**^33^ by Method C (overnight) and purified by treatment with Et_2_O, in 81% yield; ^1^H-NMR (CDCl_3_, 400 MHz) *δ* 1.55–1.70 (m, 4H, cycloheptane CH_2_), 1.75–1.90, 2.70–2.75 and 3.05–3.15 (m, each 2H, cycloheptane CH_2_), 7.40–7.55 (m, 3H, aromatic CH), 7.90–7.95 (m, 2H, aromatic CH), 12.00 (s, 1H, NH).

2-[(3-Methoxybenzoyl)amino]-5,6,7,8-tetrahydro-4*H*-cyclohepta[*b*]thiophene-3-carboxylic acid (**49**). The title compound was prepared starting from **40** by Method C (overnight) and purified by treatment with Et_2_O, in 61% yield; ^1^H-NMR (DMSO-*d*_6_, 400 MHz) *δ* 1.50–1.60 (m, 4H, cycloheptane CH_2_), 1.65–1.75, 2.75–2.85, and 3.05–3.10 (m, each 2H, cycloheptane CH_2_), 3.80 (s, 3H, OCH_3_), 7.25 (d, *J* = 8.0 Hz, 1H, aromatic CH), 7.40–7.45 (m, 1H, aromatic CH), 7.55 (t, *J* = 7.7 Hz, 1H, aromatic CH), 12.20 (s, 1H, NH), 13.50 (s, 1H, COOH).

2-[(3,4-Dimethoxybenzoyl)amino]-5,6,7,8-tetrahydro-4*H*-cyclohepta[*b*]thiophene-3-carboxylic acid (**51**). The title compound was prepared starting from **42** by Method C (12 h) and purified by treatment with Et_2_O, in 90% yield; ^1^H NMR (DMSO-*d*_6_, 400 MHz) *δ* 1.45–1.55 (m, 4H, cycloheptane CH_2_), 1.70–1.75, 2.60–2.65, and 3.00–3.05 (m, each 2H, cycloheptane CH_2_), 3.75 (s, 6H, OCH_3_), 7.10 (d, *J* = 8.1 Hz, 1H, aromatic CH), 7.35–7.40 (m, 2H, aromatic CH), 12.20 (s, 1H, NH).

2-[(3,4-Dimethoxybenzoyl)amino]-4,5,6,7-tetrahydro-1-benzothiophene-3-carboxylic acid (**52**). The title compound was prepared starting from **43** by Method C (24 h) and purified by treatment with Et_2_O, in 56% yield; ^1^H NMR (DMSO-*d*_6_, 400 MHz) *δ* 1.60–1.70 (m, 4H, cyclohexane CH_2_), 2.55–2.60 and 2.65–2.70 (m, each 2H, cyclohexane CH_2_), 3.75 (s, 6H, OCH_3_), 4.25 (q, *J* = 7.0 Hz, 2H, *CH_2_*CH_3_), 7.05–7.10 (d, *J* = 8.1 Hz, 1H, aromatic CH), 7.40–7.45 (m, 2H, aromatic CH), 12.30 (s, 1H, NH), 13.25 (bs, 1H, COOH).

2-[(3,4-Dimethoxybenzoyl)amino]-5,6-dihydro-4*H*-cyclopenta[*b*]thiophene-3-carboxylic acid (**53**). The title compound was prepared starting from **44** by Method C (24 h) and purified by treatment with Et_2_O, in 96% yield; ^1^H NMR (DMSO-*d*_6_, 400 MHz) *δ* 2.70–2.75 (m, 4H, cyclopentane CH_2_), 3.25–3.30 (m, 2H, cyclopentane CH_2_), 3.75 (s, 6H, OCH_3_), 7.05 (d, *J* = 8.5 Hz, 1H, aromatic CH), 7.40–7.45 (m, 2H, 1H, aromatic CH), 12.00 (s, 1H, NH), 13.20 (bs, 1H, COOH).

### General procedure for cyclisation (Method D)

A mixture of the appropriate thiophene-3-carboxylic acid (1.0 equiv) and acetic anhydride (10.5 equiv) was irradiated in a microwave oven at 100 °C for 30 min. The reaction mixture was then evaporated to dryness obtaining a residue, which was treated with hot petroleum ether and then purified as described below.

2–(2-Methoxyphenyl)-6,7,8,9-tetrahydro-4*H*,5*H*-cyclohepta[4,5]thieno[2,3-*d*][1,3]oxazin-4-one (**11**). The title compound was prepared starting from **45**^28^ by Method D and purified by crystallization by EtOH, in 55% yield; ^1^H-NMR (CDCl_3_, 400 MHz) *δ* 1.60–1.75 (m, 4H, cycloheptane CH_2_), 1.85–2.00, 2.75–3.00, and 3.10–3.25 (m, each 2H, cycloheptane CH_2_), 4.00 (s, 3H, OCH_3_), 6.90–7.10 (m, 2H, aromatic CH), 7.45 (dt, *J* = 5.7 and 6.5 Hz, 1H, aromatic CH), 7.85 (dd, *J* = 1.8 and 7.6 Hz, 1H, aromatic CH); ^13 ^C NMR (DMSO-*d*_6_, 101 MHz): *δ* 26.9, 27.5, 27.7, 29.3, 32.0, 55.9, 116.6, 118.2, 120.3, 122.0, 128.8, 135.3, 137.4, 139.2, 155.4, 159.4, 160.4, 163.1; HRMS: *m/z* calcd for C_18_H_17_NO_3_S 328.1008 (M + H)^+^, found 328.1005.

2–(4-Chlorophenyl)-6,7,8,9-tetrahydro-4*H*,5*H*-cyclohepta[4,5]thieno[2,3-*d*][1,3]oxazin-4-one (**13**). The title compound was prepared starting from **46**^25^ by Method D and purified by crystallization by EtOH, in 68% yield; ^1^H NMR (DMSO-*d*_6_, 400 MHz): *δ* 1.50–1.70 (m, 4H, cycloheptane CH_2_), 1.75–1.85 (m, 2H, cycloheptane CH_2_), 2.80–2.90 and 3.05–3.15 (m, each 2H, cycloheptane CH_2_), 7.55 (d, *J* = 8.6 Hz, 2H, aromatic CH), 8.05 (d, *J* = 8.6 Hz, 2H, aromatic CH), ^13 ^C NMR (DMSO-*d*_6_, 101 MHz): δ 27.0, 27.6, 27.7, 29.5, 32.0, 120.4, 129.7, 129.8, 137.6, 137.9, 139.5, 155.4, 158.8, 160.7, 162.3; HRMS: *m/z* calcd for C_17_H_14_ClNO_2_S 332.0513 (M + H)^+^, found 332.0511.

2-Phenyl-6,7,8,9-tetrahydro-4*H*,5*H*-cyclohepta[4,5]thieno[2,3-*d*][1,3]oxazin-4-one (**14**). The title compound was prepared starting from **47** by Method D and purified by crystallization by EtOH, in 68% yield; ^1^H NMR (DMSO-*d*_6_, 400 MHz): δ 1.50–1.65 (m, 4H, cycloheptane CH_2_), 1.75–1.85 (m, 2H, cycloheptane CH_2_), 2.80–2.90 and 3.05–3.15 (m, each 2H, cycloheptane CH_2_), 7.50–7.65 (m, 3H, aromatic CH), 8.05 (d, *J* = 7.4 Hz, 2H, aromatic CH); ^13 ^C NMR (DMSO-*d*_6_, 101 MHz): *δ* 27.0, 27.6, 27.8, 29.5, 32.0, 117.3, 128.0, 129.5, 129.9, 133.1, 137.5, 139.1, 155.2, 158.3, 159.8. HRMS: *m/z* calcd for C_17_H_15_NO_2_S 298.0902 (M + H)^+^, found 298.0899.

2–(2-Fluorophenyl)-6,7,8,9-tetrahydro-4*H*,5*H*-cyclohepta[4,5]thieno[2,3-*d*][1,3]oxazin-4-one (**15**). The title compound was prepared starting from **48**^25^ by Method D and purified by crystallization by petroleum ether, in 20% yield; ^1^H NMR (DMSO-*d*_6_, 400 MHz): δ 1.50–1.70 (m, 4H, cycloheptane CH_2_), 1.75–1.85 (m, 2H, cycloheptane CH_2_), 2.80–2.90 and 3.05–3.15 (m, each 2H, cycloheptane CH_2_), 6.85–6.95 (m, 2H, aromatic CH), 7.15 (t, *J* = 8.8 Hz, 2H, aromatic CH), 7.35 (q, *J* = 7.6 Hz, 2H, aromatic CH), 7.60–7.70 (m, 1H, aromatic CH), 8.00 (t, *J* = 7.7 Hz, 1H, aromatic CH); ^13 ^C NMR (DMSO-*d*_6_, 101 MHz): *δ* 27.0, 27.6, 27.7, 29.5, 32.1, 117.5, 117.7 (d, *J*_C−F_ = 21 Hz), 118.4 (d, *J*_C−F_ = 9 Hz), 125.3 (d, *J*_C−F_ = 3 Hz), 131.3, 135.0 (d, *J*_C−F_ = 9 Hz), 137.5, 139.8, 155.1, 155.8 (d, *J*_C−F_ = 5 Hz), 159.3 (d, *J*_C−F_ = 19 Hz), 162.0. HRMS: *m/z* calcd for C_17_H_14_FNO_2_S 316.0808 (M + H)^+^, found 316.0805.

2–(3-Methoxyphenyl)-6,7,8,9-tetrahydro-4*H*,5*H*-cyclohepta[4,5]thieno[2,3-*d*][1,3]oxazin-4-one (**18**). The title compound was prepared starting from **49** by Method D and purified by crystallization by EtOH, in 50% yield; ^1^H-NMR (CDCl_3_, 400 MHz) *δ* 1.70–1.80 (m, 4H, cycloheptane CH_2_), 1.89–1.95, 2.80–2.85, and 3.10–3.20 (m, each 2H, cycloheptane CH_2_), 3.85 (s, 3H, OCH_3_), 7.00–7.10 (m, 1H, aromatic CH), 7.35 (t, *J* = 8.1 Hz, 1H, aromatic CH), 7.70 (t, *J* = 2.4 Hz, 1H, aromatic CH), 7.80 (dt, *J* = 1.2 and 2.5 Hz, 1H, aromatic CH); ^13 ^C NMR (DMSO-*d*_6_, 101 MHz): *δ* 26.9, 27.5, 27.7, 29.4, 32.0, 55.7, 112.2, 117.3, 119.3, 120.4, 130.6, 131.1, 137.4, 139.2, 155.1, 158.0, 159.6, 159.9; HRMS: *m/z* calcd for C_18_H_17_NO_3_S 328.1008 (M + H)^+^, found 328.1005.

2–(4-Methoxyphenyl)-6,7,8,9-tetrahydro-4*H*,5*H*-cyclohepta[4,5]thieno[2,3-*d*][1,3]oxazin-4-one (**19**). The title compound was prepared starting from **50**^25^ by Method D and purified by crystallization by EtOH, in 44% yield; ^1^H-NMR (CDCl_3_, 400 MHz) δ 1.55–1.65 (m, 4H, cycloheptane CH_2_), 1.80–1.85, 2.80–2.85, and 3.10–3.15 (m, each 2H, cycloheptane CH_2_), 3.80 (s, 3H, OCH_3_), 7.05 and 8.05 (d, *J* = 9.1 Hz, each 2H, aromatic CH); ^13 ^C NMR (DMSO-*d*_6_, 101 MHz): δ 26.9, 27.5, 27.7, 29.3, 32.0, 55.9, 114.9, 116.6, 122.0, 130.1, 137.3, 138.1, 155.3, 158.4, 160.3, 163.2; HRMS: *m/z* calcd for C_18_H_17_NO_3_S 328.1008 (M + H)^+^, found 328.1004.

2–(3,4-Dimethoxyphenyl)-6,7,8,9-tetrahydro-4*H*,5*H*-cyclohepta[4,5]thieno[2,3-*d*][1,3]oxazin-4-one (**54**). The title compound was prepared starting from **51** by Method D and purified by treatment with Et_2_O, in 89% yield; ^1^H NMR (DMSO-*d*_6_, 400 MHz) *δ* 1.50–1.60 (m, 4H, cycloheptane CH_2_), 1.80–1.90, 2.80–2.90, and 3.10–3.20 (m, each 2H, cycloheptane CH_2_), 3.85 (s, 6H, OCH_3_), 7.05 (d, *J* = 8.6 Hz, 1H, aromatic CH), 7.50 (d, *J* = 2.0 Hz, 1H, aromatic CH), 7.70 (dd, *J* = 2.0 and 8.5 Hz, 1H, aromatic CH).

2–(3,4-Dimethoxyphenyl)-5,6,7,8-tetrahydro-4*H*-[1]benzothieno[2,3-*d*][1,3]oxazin-4-one (**55**). The title compound was prepared starting from **52** by Method D and purified by treatment with Et_2_O, in 61% yield; ^1^H NMR (DMSO-*d*_6_, 400 MHz) *δ* 1.65–1.75 and 2.65–2.75 (m, each 4H, cyclohexane CH_2_), 3.75 (s, 6H, OCH_3_), 7.05 (d, *J* = 8.6 Hz, 1H, aromatic CH), 7.55 (d, *J* = 2.0 Hz, 1H, aromatic CH), 7.70 (dd, *J* = 2.0 and 8.5 Hz, 1H, aromatic CH).

2–(3,4-Dimethoxyphenyl)-6,7-dihydro-4*H*,5*H*-cyclopenta[4,5]thieno[2,3-*d*][1,3]oxazin-4-one (**56**). The title compound was prepared starting from **53** by Method D and purified by flash chromatography eluting with CHCl_3_, in 56% yield; ^1^H NMR (CDCl_3_, 400 MHz) *δ* 2.45 (quin, *J* = 7.0 Hz, 1H, cyclopentane CH_2_), 2.95 (quin, *J* = 7.0 Hz, 2H, cyclopentane CH_2_), 3.90 and 3.95 (s, each 3H, OCH_3_), 6.85 (d, *J* = 8.5 Hz, 1H, aromatic CH), 7.70 (d, *J* = 2.0 Hz, 1H, aromatic CH), 7.90 (dd, *J* = 2.0 and 8.5 Hz, 1H, aromatic CH).

2–(3,4-Dihydroxyphenyl)-6,7,8,9-tetrahydro-4*H*,5*H*-cyclohepta[4,5]thieno[2,3-*d*][1,3]oxazin-4-one (**10**). The title compound was prepared starting from **54** by Method A and purified by crystallization by a cyclohexane/EtOAc mixture, in 65% yield; ^1^H NMR (DMSO-*d*_6_, 400 MHz) *δ* 1.60–1.70 (m, 4H, cycloheptane CH_2_), 1.80–1.90, 2.80–2.90, and 3.10–3.20 (m, each 2H, cycloheptane CH_2_), 6.90 and 7.45 (d, *J* = 8.4 Hz, each 1H, aromatic CH), 7.50 (s, 1H, aromatic CH), 9.5 and 9.95 (bs, each 1H, OH); ^13 ^C NMR (DMSO-*d*_6_, 101 MHz) δ 27.0, 27.6, 27.8, 29.4, 32.1, 115.0, 116.1, 116.3, 116.4, 120.6, 120.9, 137.2, 137.7, 146.0, 150.9, 155.4, 158.8, 160.8; HRMS: *m/z* calcd for C_17_H_15_NO_4_S 330.0801 (M + H)^+^, found 330.0809.

2–(2-Hydroxyphenyl)-6,7,8,9-tetrahydro-4*H*,5*H*-cyclohepta[4,5]thieno[2,3-*d*][1,3]oxazin-4-one (**12**). The title compound was prepared starting from **11** by Method A and purified by crystallization by EtOH, in 83% yield; ^1^H NMR (DMSO-*d*_6_, 400 MHz): δ 1.55–1.70 (m, 4H, cycloheptane CH_2_), 1.80–1.90 (m, 2H, cycloheptane CH_2_), 2.80–2.90 and 3.05–3.15 (m, each 2H, cycloheptane CH_2_), 6.90–7.05 (m, 2H, aromatic CH), 7.45 (dt, *J* = 1.6 and 7.8 Hz, 1H, aromatic CH), 7.85 (dd, *J* = 1.6 and 8.4 Hz, 1H, aromatic CH), 7.60–7.70 (m, 1H, aromatic CH), 11.50 (s, 1H, OH); ^13 ^C NMR (DMSO-*d*_6_, 101 MHz): δ 27.0, 27.6, 27.7, 29.4, 32.0, 118.1, 120.2, 128.7, 135.2, 137.5, 139.2, 155.1, 155.8, 159.4, 162.0. HRMS: *m/z* calcd for C_17_H_15_NO_3_S 314.0852 (M + H)^+^, found 314.0851.

2–(3-Hydroxyphenyl)-6,7,8,9-tetrahydro-4*H*,5*H*-cyclohepta[4,5]thieno[2,3-*d*][1,3]oxazin-4-one (**16**). The title compound was prepared starting from **18** by Method A and purified by crystallization by EtOH, in 91% yield; ^1^H NMR (DMSO-*d*_6_, 400 MHz): *δ* 1.50–1.70 (m, 4H, cycloheptane CH_2_), 1.75–1.85 (m, 2H, cycloheptane CH_2_), 2.80–2.90 and 3.05–3.15 (m, each 2H, cycloheptane CH_2_), 7.00 (dd, *J* = 2.5 and 8.2 Hz, 1H, aromatic CH), 7.35 (t, *J* = 7.9 Hz, 1H, aromatic CH), 7.45–7.55 (m, 2H, aromatic CH), 9.90 (s, 1H, OH); ^13 ^C NMR (DMSO-*d*_6_, 101 MHz): δ 27.0, 27.6, 27.7, 29.5, 32.1, 114.3, 117.3, 118.9, 120.3, 130.6, 131.0, 137.5, 139.0, 155.2, 158.2, 158.3, 159.8. HRMS: *m/z* calcd for C_17_H_15_NO_3_S 314.0852 (M + H)^+^, found 314.0855.

2–(4-Hydroxyphenyl)-6,7,8,9-tetrahydro-4*H*,5*H*-cyclohepta[4,5]thieno[2,3-*d*][1,3]oxazin-4-one (**17**). The title compound was prepared starting from **19** by Method A and purified by crystallization by EtOH, in 64% yield; ^1^H NMR (DMSO-*d*_6_, 400 MHz): *δ* 1.50–1.65 (m, 4H, cycloheptane CH_2_), 1.75–1.85 (m, 2H, cycloheptane CH_2_), 2.75–2.85 and 3.05–3.15 (m, each 2H, cycloheptane CH_2_), 6.85 (d, *J* = 8.6 Hz, 2H, aromatic CH), 7.95 (d, *J* = 8.6 Hz, 2H, aromatic CH), 10.50 (s, 1H, OH); ^13 ^C NMR (DMSO-*d*_6_, 101 MHz): *δ* 27.0, 27.6, 27.8, 29.4, 32.1, 116.3, 120.4, 130.3, 137.2, 137.8, 155.4, 158.8, 160.7, 162.3. HRMS: *m/z* calcd for C_17_H_15_NO_3_S 314.0852 (M + H)^+^, found 314.0848.

2–(3,4-Dihydroxyphenyl)-5,6,7,8-tetrahydro-4*H*-[1]benzothieno[2,3-*d*][1,3]oxazin-4-one (**20**). The title compound was prepared starting from **55** by Method A and purified by flash chromatography eluting with MeOH/CHCl_3_ (7.5%); in 30% yield; ^1^H NMR (DMSO-*d*_6_, 400 MHz) δ 1.70–1.80 and 2.70–2.80 (m, each 4H, cyclohexane CH_2_), 6.85 (d, *J* = 8.3 Hz, 1H, aromatic CH), 7.45 (dd, *J* = 2.0 and 8.3 Hz, 1H, aromatic CH), 7.50 (d, *J* = 2.0 Hz, 1H, aromatic CH), 9.5 and 9.95 (s, each 1H, OH); ^13 ^C NMR (DMSO-*d*_6_, 101 MHz) δ 21.9, 22.7, 24.8, 25.2, 115.0, 115.4, 116.4, 120.6, 121.0, 131.7, 133.6, 146.0, 151.0, 154.9, 159.1, 162.6; HRMS: *m/z* calcd for C_16_H_13_NO_4_S 316.0644 (M + H)^+^, found 316.0651.

2–(3,4-Dihydroxyphenyl)-6,7-dihydro-4*H*,5*H*-cyclopenta[4,5]thieno[2,3-*d*][1,3]oxazin-4-one (**21**). The title compound was prepared starting from **56** by Method A and purified by flash chromatography eluting with MeOH/CHCl_3_ (7.5%), in 61% yield; ^1^H NMR (DMSO-*d*_6_, 400 MHz) δ 2.35 (quin, *J* = 7.1 Hz, 1H, cyclopentane CH_2_), 2.90 (quin, *J* = 7.1 Hz, 2H, cyclopentane CH_2_), 6.85 (d, *J* = 8.3 Hz, 1H, aromatic CH), 7.45 (dd, *J* = 2.0 and 8.3 Hz, 1H, aromatic CH), 7.50 (d, *J* = 2.0 Hz, 1H, aromatic CH), 9.5 and 9.95 (s, each 1H, OH); ^13 ^C NMR (DMSO-*d*_6_, 101 MHz) δ 27.7, 28.7, 29.5, 112.8, 115.0, 116.4, 120.6, 121.0, 138.9, 140.6, 146.0, 150.9, 155.1, 158.8, 167.7; HRMS: *m/z* calcd for C_15_H_11_NO_4_S 302.0488 (M + H)^+^, found 302.0493.

Ethyl 2-[(3,4-dimethoxybenzoyl)amino]-1-benzothiophene-3-carboxylate (**57**). A mixture of **43** (0.54 g, 1.38 mmol), and DDQ (0.47 g, 2.0 mmol) in benzene (15 ml) was refluxed for 24 h. The reaction mixture was then poured into a saturated solution of NaHCO_3_ and extracted with EtOAc. The organic layers were evaporated to dryness obtaining a solid which was purified by flash chromatography eluting with EtOAc/petroleum ether (15%), to give **57** (0.5 g, 93%); ^1^H NMR (DMSO-*d*_6_, 400 MHz) δ 1.45 (t, *J* = 7.0 Hz, 3H, CH_2_*CH_3_*), 3.80 (s, 3H, OCH_3_), 4.45 (q, *J* = 7.0 Hz, 2H, *CH_2_*CH_3_), 7.15–7.50 (m, 5H, aromatic CH), 7.90 and 8.20 (d, *J* = 7.8 Hz, each 1H, aromatic CH), 12.40 (s, 1H, NH).

2-[(3,4-Dimethoxybenzoyl)amino]-1-benzothiophene-3-carboxylic acid (**58**). The title compound was prepared starting from **57** by Method C (overnight) and purified by flash chromatography eluting with MeOH/CHCl_3_ (2%), in 92% yield; ^1^H NMR (DMSO-*d*_6_, 400 MHz) δ 3.80 (s, 3H, OCH_3_), 7.15–7.45 (m, 3H, aromatic CH), 7.50–7.60 (m, 2H, aromatic CH), 7.90 (d, *J* = 7.7 Hz, each 1H, aromatic CH), 8.40 (d, *J* = 8.1 Hz, each 1H, aromatic CH), 12.40 and 12.85 (s, each 1H, NH and COOH).

2–(3,4-Dimethoxyphenyl)-4*H*-[1]benzothieno[2,3-*d*][1,3]oxazin-4-one (**59**). The title compound was prepared starting from **58** by Method D and purified by treatment with Et_2_O, in 77% yield; ^1^H NMR (DMSO-*d*_6_, 400 MHz) *δ* 3.80 (s, 3H, OCH_3_), 7.15 (d, *J* = 8.6 Hz, 1H, aromatic CH), 7.50–7.70 (m, 3H, aromatic CH), 7.80 (d, *J* = 8.5 Hz, 1H, aromatic CH), 8.15 and 8.25 (d, *J* = 7.3 Hz, each 1H, aromatic CH).

2–(3,4-Dihydroxyphenyl)-4*H*-[1]benzothieno[2,3-*d*][1,3]oxazin-4-one (**22**). The title compound was prepared starting from **59** by Method A (8 h) and purified by flash chromatography eluting with MeOH/CHCl_3_ (2%), in 80% yield; ^1^H NMR (DMSO-*d*_6_, 400 MHz) *δ* 6.95 (d, *J* = 8.2 Hz, 1H, aromatic CH), 7.50–7.70 (m, 4H, aromatic CH), 8.15 and 8.25 (d, *J* = 7.8 Hz, each 1H, aromatic CH), 9.65 and 10.20 (s, each 1H, OH); ^13 ^C NMR (DMSO-*d*_6_, 101 MHz) δ 110.4, 115.5, 116.4, 120.2, 121.9, 123.4, 123.7, 126.7, 126.9, 133.2, 134.9, 146.1, 151.8, 154.6, 161.7, 167.0; HRMS: *m/z* calcd for C_16_H_9_NO_4_S 312.0331 (M + H)^+^, found 312.0336.

2-Chlorocyclohept-1-ene-1-carbonitrile (**61**). A solution of **60**^35^ (3.15 g, 19.85 mmol) in NMP (10 ml) was added of NH_2_OH hydrochloride (1.65 g, 28.83 mmol) and it was maintained at 115 °C for 8 h. After cooling, the reaction mixture was poured into ice/water and extracted with EtOAc. The organic layers were evaporated to dryness obtaining a residue that was purified by flash chromatography eluting with EtOAc/petroleum ether (5%), to give **61** (1.5 g, 50%); ^1^H NMR (CDCl_3_, 400 MHz) *δ* 1.55–1.65 (m, 4H, cycloheptane CH_2_), 1.70–1.75 (m, 2H, cycloheptane CH_2_), 2.35–2.40 and 2.65–2.70 (m, each 2H, cycloheptane CH_2_); ^13 ^C NMR (CDCl_3_, 101 MHz) *δ* 24.4, 25.5, 30.6, 31.3, 39.4, 114.0, 118.0, 152.4.

Ethyl 3-amino-5,6,7,8-tetrahydro-4*H*-cyclohepta[*b*]thiophene-2-carboxylate (**62**). A mixture of **61** (1.5 g, 9.6 mmol) and K_2_CO_3_ (1.3 g, 9.6 mmol) in MeOH (25 ml)/THF (5 ml) mixture was added of ethyl thioglycolate (1.05 ml, 9.6 mmol) and it was refluxed for 16 h. After cooling, the reaction mixture was poured into ice/water obtaining a precipitate that was filtered to give 1.2 g of a mixture of **62** and a side product (ratio 2:1), which could not be easily separated by chromatography. While only a small sample of pure compound **62** was obtained and used for its characterisation, impure compound **62** was used in the next step without further purification; ^1^H NMR (CDCl_3_, 400 MHz) *δ* 1.30 (t, *J* = 7.0 Hz, 3H, CH_2_*CH_3_*), 1.60–1.75 (m, 4H, cycloheptane CH_2_), 1.80–1.90, 2.40–2.45, and 3.70–3.75 (m, each 2H, cycloheptane CH_2_), 4.25 (q, *J* = 7.0 Hz, 2H, CH_2_*CH_3_*), 5.45 (bs, 2H, NH_2_).

### General procedure for carbodiimide formation (method E)

A solution of the appropriate synthones (1.0 equiv) and Et_3_N (1.2 equiv) in dry CH_2_Cl_2_ was added of 3,4-dimethoxybenzoyl chloride (1.5 equiv). The reaction mixture was maintained at r.t. until no starting material was detected by TLC. After cooling, the reaction mixture was poured into ice/water and extracted with CH_2_Cl_2_. The organic layers evaporated to dryness, obtaining a residue that was purified as described below.

3-[(3,4-Dimethoxybenzoyl)amino]-5,6,7,8-tetrahydro-4*H*-cyclohepta[*b*]thiophene-2-carboxylic acid (**64**). The title compound was prepared starting from **62** by Method E (24 h) to give compound **63** (analogously to compound **62**, compound **63** was obtained in mixture with a side product that could not be purified by chromatography and was used in the next step without further purification) followed by Method C (8 h) but by applying a different workup. In particular, after cooling, the reaction mixture was poured into ice/water and extracted with EtOAc, in order to extract the side compound. The water layer was then acidified with 2 N HCl obtaining a precipitate, which was filtered and treated with Et_2_O, to give **64** (0.85 g, 23% overall yield); ^1^H NMR (CDCl_3_, 400 MHz) *δ* 1.50–1.70 (m, 4H, cycloheptane CH_2_), 1.75–1.85, 2.40–2.50, and 2.75–2.85 (m, each 2H, cycloheptane CH_2_), 3.80 (s, 6H, OCH_3_), 7.10 (d, *J* = 8.4 Hz, 1H, aromatic CH), 7.50 (s, 1H, aromatic CH), 7.65 (d, *J* = 8.4 Hz, 1H, aromatic CH), 9.80 and 12.75 (s, each 1H, NH and COOH).

2–(3,4-Dimethoxyphenyl)-7,8,9,10-tetrahydrocyclohepta[4,5]thieno[3,2-*d*][1,3]oxazin-4(6*H*)-one (**65**). The title compound was prepared starting from **64** by Method D and purified by treatment with Et_2_O, in 61% yield; ^1^H NMR (DMSO-*d*_6_, 400 MHz) δ 1.60–1.75 (m, 4H, cycloheptane CH_2_), 1.80–1.90, (m, 2H, cycloheptane CH_2_), 2.90–3.00 (m, 4H, cycloheptane CH_2_), 3.85 (s, 6H, OCH_3_), 7.15 (d, *J* = 8.6 Hz, 1H, aromatic CH), 7.70 (d, *J* = 2.0 Hz, 1H, aromatic CH), 7.80 (dd, *J* = 2.0 and 8.6 Hz, 1H, aromatic CH).

2–(3,4-Dihydroxyphenyl)-7,8,9,10-tetrahydrocyclohepta[4,5]thieno[3,2-*d*][1,3]oxazin-4(6*H*)-one (**23**). The title compound was prepared starting from **65** by Method A (6 h) and purified by flash chromatography eluting with MeOH/CHCl_3_ (2%), in 67% yield; ^1^H NMR (DMSO-*d*_6_, 400 MHz) δ 1.50–1.70 (m, 4H, cycloheptane CH_2_), 1.80–1.90, 2.80–2.85, and 2.85–2.90 (m, each 2H, cycloheptane CH_2_), 6.80 (d, *J* = 8.3 Hz, 1H, aromatic CH), 7.50 (dd, *J* = 2.1 and 8.3 Hz, 1H, aromatic CH), 7.60 (d, *J* = 2.1 Hz, 1H, aromatic CH), 9.50 and 9.90 (s, each 1H, OH); ^13 ^C NMR (DMSO-*d*_6_, 101 MHz) δ 26.2, 27.1, 27.6, 31.3, 32.2, 110.9, 115.2, 116.3, 120.9, 121.0, 137.2, 145.9, 150.9, 154.4, 155.2, 156.2, 161.6; HRMS: *m/z* calcd for C_17_H_15_NO_4_S 330.0801 (M + H)^+^, found 330.0806 (M + H+).

Ethyl 3-[(3,4-dimethoxybenzoyl)amino]-1-benzothiophene-2-carboxylate (**67**). The title compound was prepared starting from **66**^36^ by Method E (48 h) and purified by treatment with Et_2_O, in 89% yield; ^1^H NMR (CDCl_3_, 400 MHz) *δ* 1.40 (t, *J* = 7.0 Hz, 3H, CH_2_*CH_3_*), 3.95 (s, 6H, OCH_3_), 4.40 (q, *J* = 7.0 Hz, 2H, CH_2_*CH_3_*), 6.95 (d, *J* = 8.2 Hz, 1H, aromatic CH), 7.40–7.55 (m, 2H, aromatic CH), 7.65–7.80 (m, 3H, aromatic CH), 8.25 (d, *J* = 7.7 Hz, 1H, aromatic CH), 10.50 (s, 1H, NH).

3-[(3,4-Dimethoxybenzoyl)amino]-1-benzothiophene-2-carboxylic acid (**68**). The title compound was prepared starting from **67** by Method C (4 h) and purified by treatment with Et_2_O, in 58% yield; ^1^H NMR (DMSO-*d*_6_, 400 MHz) *δ* 3.90 (s, 6H, OCH_3_), 7.10 (d, *J* = 8.6 Hz, 1H, aromatic CH), 7.40–7.80 (m, 5H, aromatic CH), 8.00 (d, *J* = 7.9 Hz, 1H, aromatic CH), 10.30 (s, 1H, NH), 13.50 (bs, 1H, COOH).

2–(3,4-Dimethoxyphenyl)-4*H*-[1]benzothieno[3,2-*d*][1,3]oxazin-4-one (**69**). The title compound was prepared starting from **68** by Method D and purified by treatment with Et_2_O, in 88% yield; ^1^H NMR (DMSO-*d*_6_, 400 MHz) *δ* 3.95 and 3.90 (s, each 3H, OCH_3_), 7.20 (d, *J* = 8.6 Hz, 1H, aromatic CH), 7.60–7.80 (m, 3H, aromatic CH), 7.85 (dd, *J* = 1.9 and 8.5 Hz, 1H, aromatic CH), 8.20 (d, *J* = 8.0 Hz, 1H, aromatic CH), 8.35 (d, *J* = 7.6 Hz, 1H, aromatic CH).

2–(3,4-Dihydroxyphenyl)-4*H*-[1]benzothieno[3,2-*d*][1,3]oxazin-4-one (**24**). The title compound was prepared starting from **69** by Method A (overnight) and purified by flash chromatography eluting with MeOH/CHCl_3_ (2%), in 38% yield; ^1^H NMR (DMSO-*d*_6_, 400 MHz) δ 6.95 (d, *J* = 8.3 Hz, 1H, aromatic CH), 7.60–7.75 (m, 4H, aromatic CH), 8.20 and 8.30 (d, *J* = 8.0 Hz, each 1H, aromatic CH), 9.60 and 10.10 (s, each 1H, OH); ^13 ^C NMR (DMSO-*d*_6_, 101 MHz) *δ* 115.0, 115.3, 116.3, 120.6, 121.4, 124.1, 124.5, 126.4, 130.5, 133.3, 141.9, 146.0, 151.2, 153.0, 156.1, 161.8; HRMS: *m/z* calcd for C_16_H_9_NO_4_S 312.0331 (M + H)^+^, found 312.0336.

Ethyl 2-aminocyclohept-1-ene-1-carboxylate (**71**). A mixture of **70**^37^ (1.0 g, 5.4 mmol) and ammonium acetate (2.0 g, 27.1 mmol) in MeOH (15 ml) was maintained at r.t. overnight. Then, the reaction mixture was evaporated to dryness, obtaining a white solid, which was treated with CH_2_Cl_2_, filtered, and washed with ample CH_2_Cl_2_. The combined CH_2_Cl_2_ was washed with water and evaporated to dryness, to give **71** (0.88 g, 88%); ^1^H NMR (CDCl_3_, 400 MHz) *δ* 1.25 (t, *J* = 7.0 Hz, 3H, CH_2_*CH_3_*), 1.40–1.50 (m, 2H, cycloheptane CH_2_), 1.60–1.70 (m, 4H, cycloheptane CH_2_), 2.25–2.30 and 2.45–2.50 (m, each 2H, cycloheptane CH_2_), 4.10 (q, *J* = 7.0 Hz, 2H, CH_2_*CH_3_*).

Ethyl 2-[(3,4-dimethoxybenzoyl)amino]cyclohept-1-ene-1-carboxylate (**72**). The title compound was prepared starting from **71** by Method E (24 h) and purified by flash chromatography eluting with EtOAc/petroleum ether (5%), in 32% yield; ^1^H NMR (CDCl_3_, 400 MHz) *δ* 1.25 (t, *J* = 7.0 Hz, 3H, CH_2_*CH_3_*), 1.50–1.60 (m, 2H, cycloheptane CH_2_), 1.70–1.80 (m, 4H, cycloheptane CH_2_), 2.55–2.60 and 3.20–3.25 (m, each 2H, cycloheptane CH_2_), 3.90 (s, 6H, OCH_3_), 4.20 (q, *J* = 7.0 Hz, 2H, CH_2_*CH_3_*), 6.90–6.95 (m, 1H, aromatic CH), 7.55–7.60 (m, 2H, aromatic CH), 12.25 (bs, 1H, NH).

2-[(3,4-Dimethoxybenzoyl)amino]cyclohept-1-ene-1-carboxylic acid (**73**). The title compound was prepared starting from **72** by Method C (overnight) and purified by treatment with Et_2_O, in 100% yield; ^1^H NMR (DMSO-*d*_6_, 400 MHz) *δ* 1.40–1.50 (m, 2H, cycloheptane CH_2_), 1.55–1.75 (m, 4H, cycloheptane CH_2_), 2.45–2.50 and 3.05–3.10 (m, each 2H, cycloheptane CH_2_), 3.75 (s, 6H, OCH_3_), 7.05 (d, *J* = 8.3 Hz, 1H, aromatic CH), 7.40–7.50 (m, 2H, aromatic CH), 12.10 (s, 1H, NH).

2–(3,4-Dimethoxyphenyl)-6,7,8,9-tetrahydrocyclohepta[*d*][1,3]oxazin-4(5*H*)-one (**74**). The title compound was prepared starting from **73** by Method D and purified by treatment with Et_2_O, in 35% yield; ^1^H NMR (DMSO-*d*_6_, 400 MHz) *δ* 1.50–1.65 (m, 4H, cycloheptane CH_2_), 1.70–1.80 (m, 2H, cycloheptane CH_2_), 2.60–2.65 and 2.70–2.75 (m, each 2H, cycloheptane CH_2_), 3.80 (s, 6H, OCH_3_), 7.10 (d, *J* = 8.6 Hz, 1H, aromatic CH), 7.55 (d, *J* = 2.0 Hz, 1H, aromatic CH), 7.70 (dd, *J* = 2.0 and 8.6 Hz, 1H, aromatic CH),

2–(3,4-Dihydroxyphenyl)-6,7,8,9-tetrahydrocyclohepta[*d*][1,3]oxazin-4(5*H*)-one (**25**). The title compound was prepared starting from **74** by Method A (overnight) and purified by flash chromatography eluting with MeOH/CHCl_3_ (2%), in 39% yield; ^1^H NMR (DMSO-*d*_6_, 400 MHz) δ 1.50–1.65 (m, 4H, cycloheptane CH_2_), 1.70–1.80 (m, 2H, cycloheptane CH_2_), 2.60–2.65 and 2.70–2.75 (m, each 2H, cycloheptane CH_2_), 6.80 (d, *J* = 8.3 Hz, 1H, aromatic CH), 7.45 (d, *J* = 8.3 Hz, 1H, aromatic CH), 7.50 (s, 1H, aromatic CH), 9.50 and 9.90 (s, each 1H, OH); ^13 ^C NMR (DMSO-*d*_6_, 101 MHz) δ 24.5, 24.8, 26.0, 31.6, 37.4, 115.0, 115.8, 116.3, 119.7, 120.8, 145.9, 151.0, 160.1, 160.6, 167.3; HRMS: *m/z* calcd for C_15_H_15_NO_4_ 274.1080 (M + H)^+^, found 274.1085.

2–(3,4-Dihydroxyphenyl)-4*H*-3,1-benzoxazin-4-one (**26**). The title compound was prepared starting from **75**^38^ by Method A, with the exception that toluene was used as a solvent and the reaction was refluxed for 1 h. The compound was purified by flash chromatography eluting with MeOH/CHCl_3_ (2%) and obtained in 24% yield; ^1^H NMR (DMSO-*d*_6_, 400 MHz) δ 6.85 (d, *J* = 8.3 Hz, 1H, aromatic CH), 7.45–7.50 and 7.55–7.60 (m, each 2H, aromatic CH), 7.85 (dt, *J* = 1.2 and 8.3 Hz, 1H, aromatic CH), 8.05 (dd, *J* = 1.2 and 7.8 Hz, 1H, aromatic CH), 9.50 and 9.90 (s, each 1H, OH); ^13 ^C NMR (DMSO-*d*_6_, 101 MHz) *δ* 115.2, 116.2, 116.8, 120.9, 121.1, 126.9, 128.2, 128.4, 137.2, 145.9, 147.2, 150.8, 157.1, 159.9; HRMS: *m/z* calcd for C_14_H_9_NO_4_ 256.0611 (M + H)^+^, found 256.0618.

2–(3,4-Dihydroxyphenyl)quinazolin-4(3*H*)-one (**27**). A mixture of 2-aminobenzamide (0.5 g, 3.6 mmol), 3,4-dihydroxybenzaldehyde (0.6 g, 4.4 mmol), and I_2_ (1.2 g, 4.7 mmol) in CH_3_CN (25 ml) was maintained at r.t. overnight. Then, the reaction mixture was poured into a solution of 5% Na_2_S_2_O_3_ obtaining a precipitate that was filtered and purified by flash chromatography eluting with MeOH/CHCl_3_ (5%), to give **27** (0.23 g, 22%); ^1^H NMR (DMSO-*d*_6_, 400 MHz) δ 6.80 (d, *J* = 8.3 Hz, 1H, aromatic CH), 7.40 (t, *J* = 7.5 Hz, 1H, aromatic CH), 7.50 (dd, *J* = 2.0 and 8.3 Hz, 1H, aromatic CH), 7.60–7.65 (m, 2H, aromatic CH), 7.70–7.80 (m, 1H, aromatic CH), 8.05 (d, *J* = 7.5 Hz, 1H, aromatic CH), 9.30 and 9.65 (bs, each 1H, OH), 12.20 (s, 1H, NH); ^13 ^C NMR (DMSO-*d*_6_) δ 115.5, 115.6, 119.9, 120.9, 123.9, 126.1, 126.2, 127.5, 134.8, 145.7, 149.4, 152.5, 162.6, 165.1; HRMS: *m/z* calcd for C_14_H_10_N_2_O_3_ 255.0770 (M + H)^+^, found 255.0778.

### HIV-1 RT expression and purification: HIV-1 RT group M subtype B

Heterodimeric RT was expressed essentially as described^19^^,^^39^. Using a BioLogic LP system (Biorad), using a combination of immobilised metal affinity (Ni-Sepharose high performance, Healthcare Lifescience) and ion exchange chromatography (Hi-trap heparin HP GE). The protein was dialysed and stored in buffer containing 50 mM Tris HCl pH 7.0, 25 mM NaCl, 1 mM EDTA, and 50% glycerol. Catalytic activities and protein concentrations were determined. Enzyme-containing fractions were pooled and aliquots were stored at −80 °C.

### HIV-1 DNA polymerase-independent RNase H activity determination

HIV RT-associated RNase H activity was measured as described^40^ using as a control the RNase H selective inhibitor RDS1759^9^, and using different amount of enzymes according to a linear range of dose-response curve: 20 ng of WT RT; 75 ng V108F RT; 50 ng Y188A RT; 30 ng W229A RT; 100 ng A502F RT.

### HIV-1 RNA-dependent DNA polymerase activity determination

RNA-dependent DNA polymerase (RDDP) activity was measured as described^17^ using and different amounts of enzymes according to a linear range of dose-response curve: 6 ng wt RT; 19 ng V108F RT; 45 ng Y188A RT; 30 ng W229A RT; A502F RT. The Yonetani-Theorell analysis was performed as previously described^41^^,^^42^.

### Site-directed mutagenesis

The QuikChange mutagenesis kit (Agilent Technologies Inc., Santa Clara, CA)was used to introduce amino acid substitutions into the p66 HIV-1 RT subunit coded in a p6HRT-prot plasmid by following the manufacturer’s instructions.

### Detection of protein inhibitor interactions by differential scanning fluorimetry

Differential Scanning Fluorimetry assay was used to determine the thermal stability of the protein according to Nettleship *et al.*^43^. In a MJ Research Opticon 2 qPCR (Bio-Rad) 96-well plate 100 ng/µl of HIV-1 RT enzyme was incubated alone or in presence of increasing concentrations of compound **22** or **24**, using the interface inhibitor compound **76**^44^ as a control in 50 µl of final volume of reaction buffer containing 20 mM HEPES, pH 7.5, 10 mM MgCl_2_, 100 mM NaCl, and a 1:1000 dilution of Sypro Orange dye (Invitrogen). The mixture was heated from 30 to 90 °C in increments of 0.2 °C. Fluorescence intensity was measured using excitation and emission wavelengths of 490 and 565 nm, respectively. Changes in protein thermal stability (ΔTm) upon inhibitor binding were analysed using the Opticon Monitor^™^ Software. All assays were performed in triplicate.

### Interaction between Mg^2+^ and HIV-1 RT inhibitors

The effects of the magnesium ions were evaluated by mean of spectrophotometric method as reported^45^, using a Ultrospec 2100 pro UV-Vis spectrophotometer (Amersham Biosciences) and Hellma quartz cuvette with 1 cm optical path. MgCl_2_ 1 M solution was prepared from powder (Carlo Erba Reagents, Milano, Italy). Each studied compound was dissolved in DMSO to a final concentration of 10 mM and diluted in milliQ water to a final concentration of 100 µM. Each obtained solution was placed in a cuvette and the UV-Vis and the spectra were recorded between 200 and 700 nm using water milliQ (with a percentage of DMSO equal to the one present in the sample solution) as reference. Thereafter, small volumes of MgCl_2_ aqueous solution were added both in the sample and in the reference cuvettes to obtain a series of solutions containing increasing concentrations of MgCl_2_, up to 1.25 mM, carefully pipetting to mix, and the UV-Vis spectra were repeated. Each experiment was performed in triplicate.

### In vitro antiviral assays

The evaluation of the antiviral activity of the target compounds against HIV-1 strain III_B_ in MT-4 cells was performed using the MTT assay as previously described^46^^,^^47^.

### Ligand preparation

Theoretical 3 D model of compound **22** was built with the Maestro software^48^. Then, the compound was subjected to a conformational search protocol with MacroModel version 9.2^49^, considering Merck molecular force fields (MMFFs)^50^ as force field and the implicit solvation model Generalised Born/Surface Area (GB/SA) water^51^. All conformational search parameters were left as default. Therefore, the compound geometry was energy minimised using Polak-Ribier Conjugate Gradient (PRCG) method, 5000 iterations and a convergence criterion of 0.05 kcal/(mol Å).

### Protein preparation

The coordinates for RTs were taken from the RCSB Protein Data Bank^52^ (PDB codes 1vrt^53^, 2zd1^54^, 1ep4^55^, 3qo9^54^, 1rti^53^, 1tv6^56^, 3lp2^57^. The proteins were prepared by means of Maestro Protein Preparation Wizard default setting. Original water molecules were removed. A502F-RT mutated enzyme was generated starting from wt protein and optimized^17^.

### Docking protocol

Molecular docking studies were performed using QMPL workflow protocol^58^^,^^59^ applying the same settings previously reported^19^. The same protocol was applied for all simulations.

### Post-docking protocol

In order to better take into account the induced fit phenomenon occurring at the ligand binding domain, the most energy favoured generated complexes were fully optimised with 10,000 steps of the Polak-Ribier conjugate gradient (PRCG) minimisation method considering OPLS_2005 force field and GB/SA implicit water. The optimisation process was performed up to the derivative convergence criterion equal to 0.1 kJ/(mol*Å).

### Graphics

The resulting complexes were considered for the binding modes graphical analysis with Pymol^60^ and Maestro Ligand Interaction visualization^48^.

## Results and discussion

### Design

Compound **1** and most of the cHTCs previously reported were characterised by two aromatic rings at both C-2 and C-3 positions of the cHTC scaffold, but they could not be essential for the activity as compared to known inhibitors^29^. Thus, the two aromatic moieties were alternatively removed by exploring substituents endowed with different electronic and steric characters (see Table 1 for the structures). In particular, by removing the catechol ring and fixing the amino group at the C-2 position, the C-3 pyridine moiety was maintained in compound **2** and then replaced by *o*-methoxyphenyl, *o*-hydroxyphenyl, and *p*-chlorophenyl rings (compounds **3–5**, respectively). The same three substituents along with the catechol moiety were explored as C-2 substituents in 3-carboxamide derivatives **6–9**. Then, the presence of only one aromatic ring was also studied in cycloheptathienooxazinone (**cHTO**) derivatives **10–13**, a set of rigid analogues.

**Table 1.** Effect of cHTC and cHTO derivatives on the HIV-1 RT-associated RNase H and RDDP functions.

^a^IC_50_: compound concentration required to inhibit the HIV-1 RT-associated RNase H activity by 50%. The table reports the average and standard deviation of three independent experiments, made in duplicate.

^b^IC_50_: compound concentration required to inhibit the HIV-1 RT-associated RNA dependent DNA polymerase activity by 50%. The table reports the average and standard deviation of three independent experiments, made in duplicate;

^c^Percentage of residual enzymatic activity in the presence of 100 µM of compound.

The biological evaluation of the three series of derivatives confirmed the catechol moiety as particularly useful to impart RNase H inhibitory activity, especially when placed at C-2 position of the cHTO scaffold as in compound **10** (IC_50_ = 1.1 μM). Given the good anti-RNase H activity of **10**, the cHTO core was maintained in a successive set of derivatives (compounds **14–19**, Table 1). In particular, over the non-substituted phenyl ring (compound **14**), the 2-fluorine derivative **15** was prepared. Moreover, based on the anti-RNase H activity shown by the *o*-hydroxy derivative **12** (IC_50_=18.2 μM), the *meta*- and *para*-hydroxyphenyl derivatives **16** and **17** were synthesised, also evaluating the methoxy analogues **18** and **19**, respectively.

Since the biological evaluation confirmed the catechol derivative **10** as the most potent in inhibiting the RNase H function within the cHTO series, further compounds (derivatives **20–27**, Table 2) were synthesised by fixing the catechol moiety at C-2 position of the oxazinone ring, while widely modifying the cycloheptathieno portion. In particular, the cycloheptane ring was reduced to a six- and five-membered rings in compounds **20** and **21**, respectively, and was also replaced by a benzene ring (compound **22**). Then, the cycloheptathiophene and benzothiophene moieties were reversed in the cycloeptathieno[3,2-*d*]oxazinone and benzothieno[3,2-*d*]oxazinone derivatives **23** and **24**, respectively. Moreover, the size of the scaffold was further reduced by deleting the thiophene ring, as in bicyclic derivatives **25** and **26**. In the case of compound **26**, the corresponding derivative **27** was also synthesised by replacing the oxygen with a nitrogen atom.

**Table 2.** Effect of oxazinone-based derivatives on the HIV-1 RT-associated RNase H and RDDP functions.

^a^IC_50_: compound concentration required to inhibit the HIV-1 RT-associated RNase H activity by 50%. The table reports the average and standard deviation of three independent experiments, made in duplicate.

^b^IC_50_: compound concentration required to inhibit the HIV-1 RT-associated RNA dependent DNA polymerase activity by 50%. The table reports the average and standard deviation of three independent experiments, made in duplicate.

### Chemistry

**Scheme 1.**
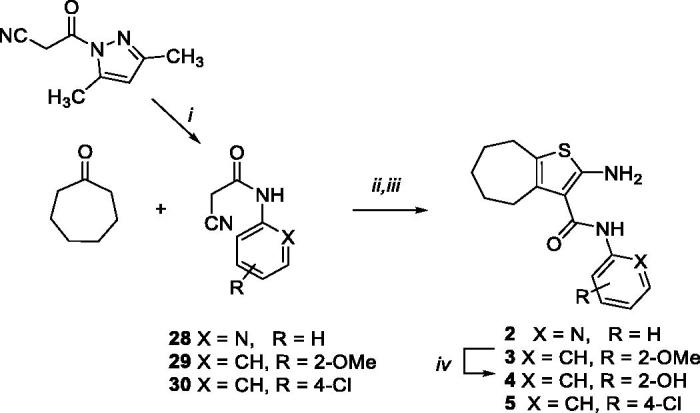
Synthetic pathway for preparation of **2–5**. Reagents: (i) suitable amine, toluene, reflux; (ii) ammonium acetate, glacial acetic acid, benzene, reflux; (iii) sulphur, *N*,*N*-diethylamine, EtOH, 40–50 °C; (iv) BBr_3_, dry CH_2_Cl_2_, rt.

The synthesis of the cHTC derivatives **2**^25^, **3**^31^, **4**, and **5**^28^, was accomplished as reported in Scheme 1, by applying a Knoevenagel condensation. In particular, reaction of cycloheptanone with 2-cyanoacetamides **28**^61^, **29**^62^, and **30**^62^, prepared by reaction of 3–(3,5-dimethyl-1*H*-pyrazol-1-yl)-3-oxopropanenitrile^61^ with the appropriate amines in toluene, followed by cyclisation performed in the presence of sulphur and *N,N*-diethylamine in EtOH, provided compounds **2**, **3**, and **5**. Compound **4** was obtained by *O*-demethylation of the methoxy derivative **3**.

**Scheme 2.**
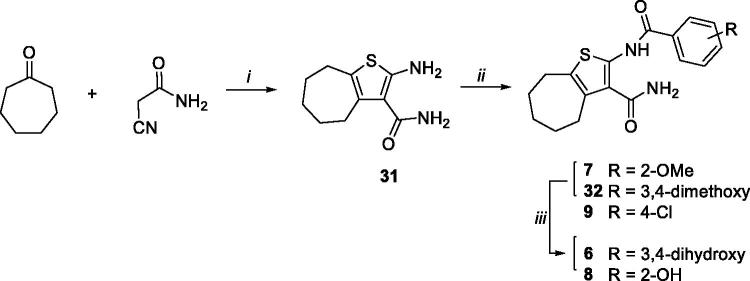
Synthetic pathway for preparation of **6–9**. Reagents: (i) sulphur, *N,N*-diethylamine, EtOH, rt; (ii) suitable benzoyl chloride, dry pyridine, rt; (iii) BBr_3_, dry CH_2_Cl_2_, rt.

Starting from synthone **31**^32^, synthesised by reacting cycloheptanone with 2-cyanoacetamide, the coupling reaction with the appropriate benzoyl chloride led to the target compounds **7** and **9**, and intermediate **32**, of which methoxy derivatives **7** and **32** were *O*-demethylated yielding hydroxy compounds **6** and **8** (Scheme 2).

**Scheme 3.**
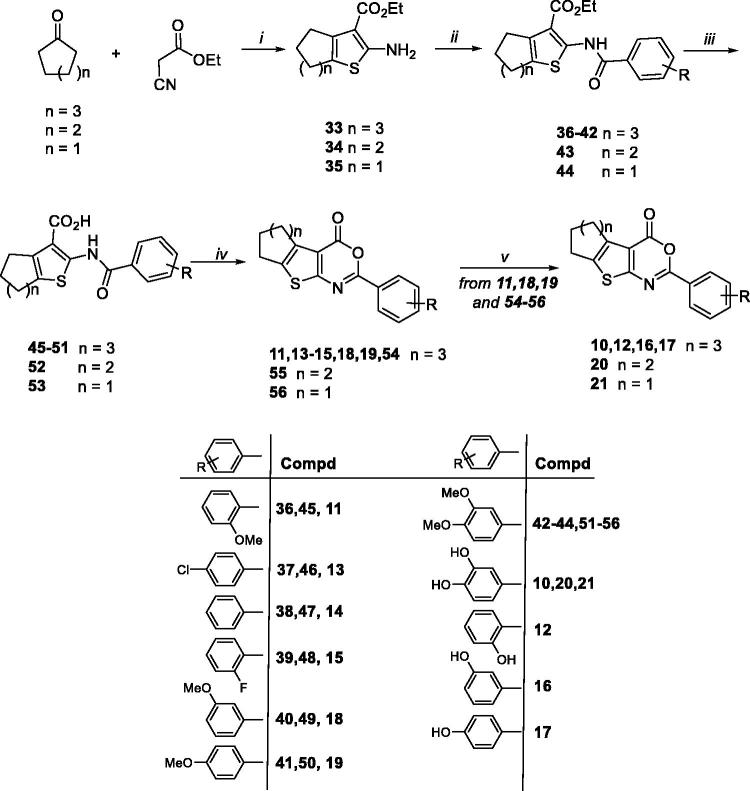
Synthetic pathway for preparation of **11–21**. Reagents: (i) sulphur, *N,N*-diethylamine, EtOH, rt; (ii) suitable benzoyl chloride, dry pyridine, rt; (iii) LiOH, H_2_O/THF (1:1), 50 °C; (iv) acetic anhydride, 100 °C MW; (v) BBr_3_, dry CH_2_Cl_2_, rt.

The synthesis of cycloheptathieno[2,3-*d*][1,3]oxazin-4-one derivatives **10–19**, tetrahydrobenzothieno[2,3-*d*][1,3]oxazin-4-one **20**, and cyclopentathieno[2,3-*d*][1,3]oxazin-4-one **21** was accomplished, as outlined in Scheme 3, starting from synthones **33**^33^, **34**^34^, and **35**^34^, respectively. Synthones **33–35** were obtained by applying the one-pot Gewald reaction by reacting the appropriate cycloalkyl ketones with ethyl cyanoacetate in the presence of sulfur and *N,N*-diethylamine in EtOH. Coupling reaction of synthones **33–35** with the appropriate acyl chlorides in pyridine furnished intermediates **36**^28^, **37**^33^, **38**^33^, **39**^25^, **40**, **41**^25^, and **42–44**, which were then hydrolysed under basic conditions to give corresponding acids **45**^28^, **46**^25^, **47**, **48**^25^, **49**, **50**^25^, and **51–53**. The successive cyclisation in acetic anhydride at 100 °C under MW irradiations furnished compounds **11**, **13–15**, **18**, **19**, and **54–56**. *O*-demethylation of monomethoxy derivatives **11**, **18**, **19** and dimethoxy derivatives **54–56** by using BBr_3_ in CH_2_Cl_2_ gave hydroxy derivatives **10**, **12**, **16**, **17**, **20**, and **21**.

**Scheme 4.**
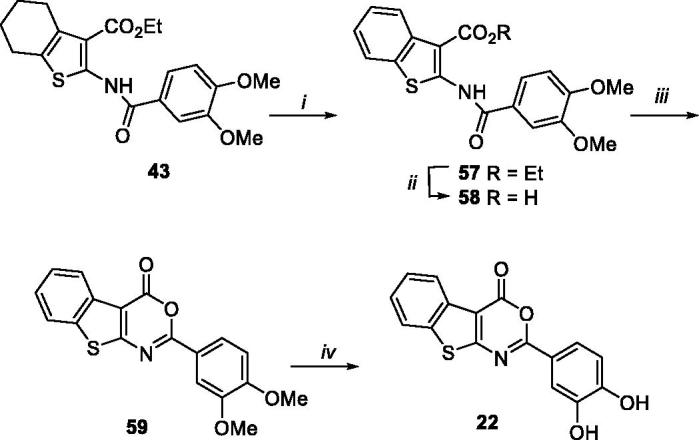
Synthetic pathway for preparation of **22**. Reagents: (i) DDQ, benzene, reflux; (ii) LiOH, H_2_O/THF (1:1), 50 °C; (iii) acetic anhydride, 100 °C MW; (iv) BBr_3_, dry CH_2_Cl_2_, rt.

Benzothieno[2,3-*d*][1,3]oxazin-4-one derivative **22** was synthesised, as reported in Scheme 4, starting from tetrahydrobenzothieno intermediate **43**, which was oxidised by using 2,3-dichloro-5,6-dicyano-1,4-benzoquinone (DDQ) in benzene to give benzothieno derivative **57**. The successive basic hydrolysis of compound **57** to intermediate **58**, cyclisation to compound **59**, and demethylation, furnished target compound **22**.

**Scheme 5.**
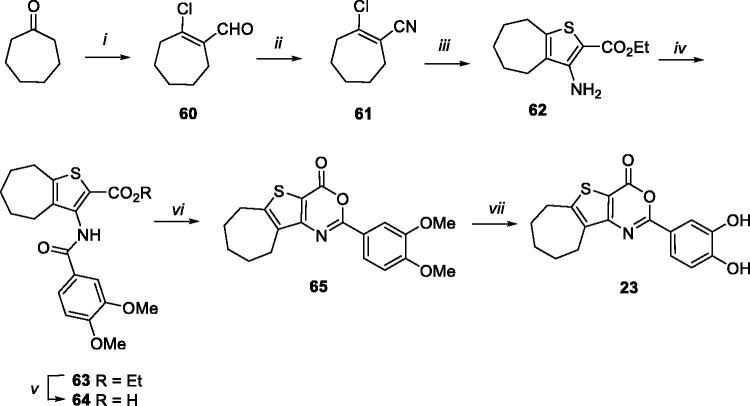
Synthetic pathway for preparation of **23**. Reagents: (i) DMF, POCl_3_, from 0 °C to rt; (ii) NMP, NH_2_OH hydrochloride, 115 °C; (iii) K_2_CO_3_, MeOH/THF (5:1), ethyl thioglycolate, reflux; (iv) 3,4-dimethoxybenzoyl chloride, Et_3_N, dry CH_2_Cl_2_, rt; (v) LiOH, H_2_O/THF (1:1), 50 °C; (vi) acetic anhydride, 100 °C MW; (vii) BBr_3_, dry CH_2_Cl_2_, rt.

The synthesis of cycloheptathieno[3,2-*d*][1,3]oxazin-4-one derivative **23** entailed the preparation of key intermediate **62**, as shown in Scheme 5. In particular, cycloheptanone was reacted with DMF and POCl_3_ to give intermediate **60**^35^, which was used for the preparation of compound **61** by reaction with hydroxylamine hydrochloride in *N*-methyl-2-pyrrolidinone (NMP). Intermediate **61** was then reacted with ethyl thioglycolate in presence of K_2_CO_3_ in a MeOH/THF mixture to give synthone **62**. Coupling reaction of **62** with 3,4-di-methoxybenzoyl chloride in CH_2_Cl_2_ in presence of Et_3_N furnished intermediate **63**, which was in turn hydrolysed, cyclized, and demethylated to target compound **23**, through intermediates **64** and **65**.

**Scheme 6.**
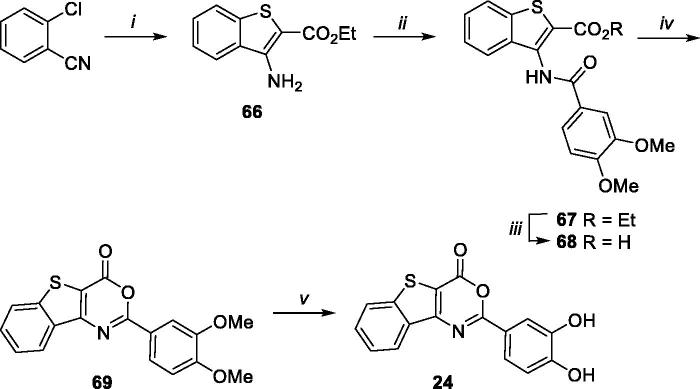
Synthetic pathway for preparation of **24**. Reagents: (i) ethyl thioglycolate, DMF, KOH, from 0 °C to 80 °C; (ii) 3,4-dimethoxybenzoyl chloride, Et_3_N, dry CH_2_Cl_2_, rt; (iii) LiOH, H_2_O/THF (1:1), 50 °C; (iv) acetic anhydride, 100 °C MW; (v) BBr_3_, dry CH_2_Cl_2_, rt.

By using an analogous procedure, benzothieno[3,2-*d*][1,3]oxazin-4-one derivative **24** was synthesised starting from synthone **66** (Scheme 6), prepared as reported in literature^36^ by reaction of 2-chlorobenzonitrile and ethyl thioglycolate in presence of KOH in DMF. Coupling reaction of synthone **66** with 3,4-dimethoxybenzoyl chloride, basic hydrolysis, cyclisation, and demethylation, furnished target compound **24**, through intermediates **67–69**.

**Scheme 7.**
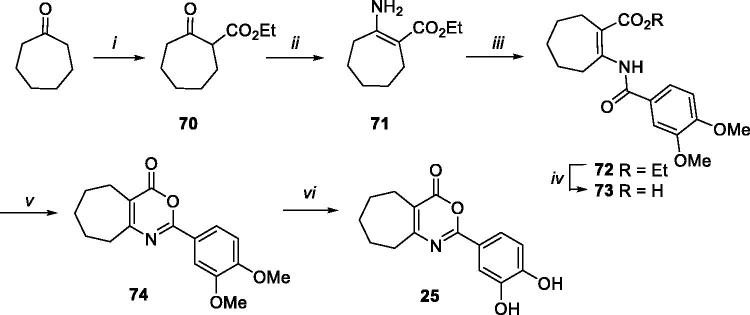
Synthetic pathway for preparation of **25**. Reagents: (i) NaH, diethyl carbonate, toluene, from 0 °C to 100 °C; (ii) ammonium acetate, MeOH, rt; (iii) 3,4-dimethoxybenzoyl chloride, Et_3_N, dry CH_2_Cl_2_, rt; (iv) LiOH, H_2_O/THF (1:1), 50 °C; (v) acetic anhydride, 100 °C MW; (vi) BBr_3_, dry CH_2_Cl_2_, rt.

Cyclohepta[*d*][1,3]oxazin-4-one derivative **25** was synthesised, as reported in Scheme 7, starting from compound **70**^37^, which was prepared by reaction of cycloheptanone and diethyl carbonate in presence of NaH in toluene. Reaction of intermediate **70** with ammonium acetate in MeOH furnished compound **71**, which in turn was subjected to coupling reaction with 3,4-dimethoxybenzoyl chloride, basic hydrolysis, cyclisation, and demethylation, furnishing target compound **25**, through intermediates **72–74**.

**Scheme 8.**
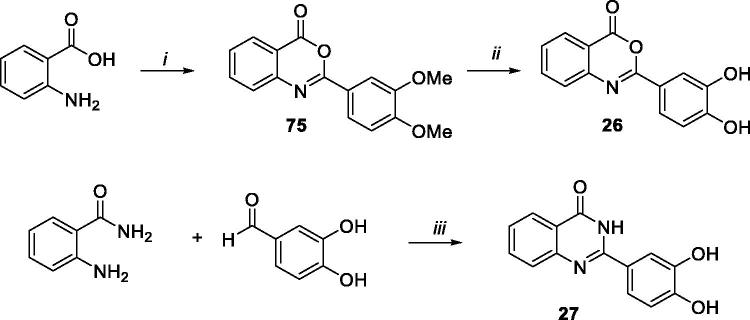
Synthetic pathway for preparation of **26** and **27**. Reagents: (i) 3,4-dimethoxybenzoyl chloride, dry pyridine, rt; (ii) BBr_3_, toluene, reflux; (iii) I_2_, CH_3_CN, rt.

As reported in Scheme 8, benzoxazin-4-one derivative **26** was synthesised by demethylation of intermediate **75**^38^, which was obtained by reaction of 2-aminobenzoic acid and 3,4-dimethoxybenzoyl chloride in pyridine. On the other hand, quinazolin-4-one **27** was synthesised, differently from the procedure reported in literature^63^, by reaction of 2-aminobenzamide with 3,4-dihydroxybenzaldehyde in presence of I_2_ in CH_3_CN (Scheme 8).

### Inhibition of RT-associated activities

The whole set of derivatives were initially evaluated for their ability to inhibit the HIV-1 RNase H function (Table 1 and Table 2). Table 1 showed that the mono-aromatic substituted derivatives are in general less potent than the previous di-substituted cHTCs^24^. However, compounds **5** (IC_50_=16.5 µM), **6** (IC_50_=2.60 µM), and **7** (IC_50_=14.0 µM) showed a good activity with the catechol 3-carboxamide derivative **6** that emerged as the most active. With the only exception of *o*-methoxyphenyl derivative **11** and *p*-chlorophenyl derivative **13**, all the tricyclic cHTOs showed RNase H inhibitory activity with IC_50_ values ranging from 1.10 and 34.9 µM. Once again, the presence of the catechol moiety granted the best anti-RNase H activity, with derivative **10** that showed an IC_50_ of 1.10 µM.

The suitability of this peculiar moiety was further confirmed and even improved by the successive series of derivatives, all bearing the catechol moiety at C-2 position of different oxazinone-based scaffolds (Table 2). In particular, when compared to compound **10**, the reduction of cycloheptane ring to cyclohexane (compound **20**) slightly decreased the anti-RNase H activity (IC_50_=3.35 µM), while its further reduction to cyclopentane (compound **21**) permitted to maintain the same biological activity (IC_50_=1.00 µM). The replacement of the cycloheptane with a benzene ring improved the activity, as shown by compound **22** that emerged as the most potent derivative of the series, with IC_50_ of 0.53 µM. When the geometry of the core was changed by reversing the thiophene portion in compound **23**, a slight decreased anti-RNase H activity emerged, while the aromatic derivative **24** showed a potent inhibitory activity (IC_50_=0.96 µM) similarly to its analogue **22**. These results suggested how the geometry of the scaffold does not influence the interaction with the target and above all that the aromatic derivatives are more suitable inhibitors than the corresponding cycloheptathiophene analogues. For the bicyclic derivatives **25** and **26**, a reduced ability to inhibit the RNase H function was observed. Finally, quinazolinone derivative **27** showed an IC_50_ comparable to that of the benzoxazine analogue **26**, indicating that the nature of the heteroatom does not play essential role in the interaction to the target domain.

From the biological evaluation of the oxazinone-based compounds, clear SAR insights emerged: (i) the catechol resulted in a critical moiety to achieve potent anti-RNase H activity; (ii) the modifications made on the scaffold, such as size reduction, geometry inversion, and nature of heteroatom, seem to be not critical for the target binding; (iii) benzene derivatives emerged more active than the cycloheptane counterparts.

To examine their specificity of action, all the compounds were tested against the RDDP function, using as a positive control the non-nucleoside RT inhibitor (NNRTI) **efavirenz** (Table 1 and Table 2). From this assay, some active compounds emerged along with others that did not recognise this RT function at all, leading to add another clear structural information. Indeed, all the catechol derivatives characterised by a tricyclic scaffold (compounds **10** and **20–24**) showed the ability to inhibit also the RDDP activity resulting dual-acting compounds. Derivative **22** emerged again the best inhibitor with an IC_50_ of 2.90 µM. Interestingly, its structural isomer **24**, which exhibited a similar anti-RNase H potency, was significantly less potent against the RDDP function (IC_50_=14.6 µM) suggesting that the RDDP binding is more susceptible to the molecular geometry. On the other hand, the bicyclic catechol derivatives **25–27** were completely inactive against RDDP, thus emerging as selective RNHIs.

### Mode of action studies

#### Investigation of Magnesium-Complexation

**Figure 2.**
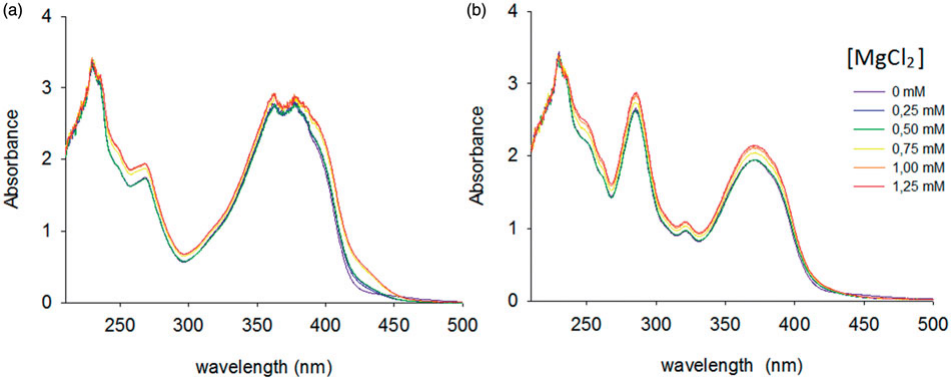
Investigation of Magnesium-Complexation. UV–vis spectra of compounds (a) **22** and (b) **24** measured alone or in the presence of increasing concentration of MgCl_2_.

Firstly, the involvement of the Mg^2+^ cofactors in the mechanism of inhibition of the new RNHIs was studied by recording the UV spectra for compound **22** together with the structurally-related **24**, alone and in the presence of increasing concentrations of MgCl_2_ (Figure 2(A) and B). Results showed that, differently from RNase H active site inhibitors^8^, compounds **22** and **24** did not show any shift in the maximum of absorbance (hypsochromic effect), neither the presence of an isosbestic point. In both cases, we observed a slight increase in absorbance across the analysed UV-Vis spectra for concentrations of MgCl_2_ equal or greater than 750 μM, which could be attributed to the moderate increase of the ionic strength of the solution.

#### Differential scanning fluorimetry

**Figure 3.**
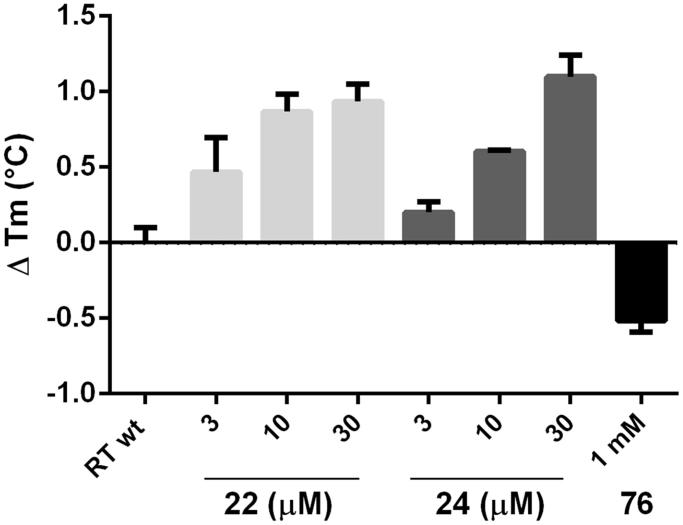
Effect of compounds **22**, **24**, and reference **76** on the thermal stability of p66/p51 HIV-1 RT.

The interaction between RT and an inhibitor can be investigated by differential scanning fluorimetry since RT inhibitors can either stabilise or destabilise the RT heterodimer^64^ so causing either an increase or a decrease in its melting temperature (Tm)^65^. In particular, compounds reported to bind close to the interface between the two subunits have been shown to negatively impact on RT Tm^44^. Hence, to investigate the effect of our compounds on the RT stability, differential scanning fluorimetry analysis was performed in presence of compounds **22** and **24**, using the previously reported interface inhibitor benzyl 2–(9-(4-ethoxyphenyl)-1,7-dimethyl-2,4-dioxo-1,4,4a,6,7,8,9,10a-octahydropyrimido[2,1-*f*]purin-3(2*H*)-yl)acetate (compound **76**)^44^ as control (Figure 3). A dose-dependent increase in the RT Tm was observed in presence of increasing concentration of both the compounds, differently to compound **76** that decreased the Tm. These data clearly indicated that the nature of the interactions occurring in the RT-inhibitor complex does not destabilise the RT conformation.

### Docking studies

In order to investigate the possible binding mode of the newly identified compounds, derivative **22** was subjected to blind docking studies by using the whole structure of the wt HIV-1 RT. In particular, the QM-polarized ligand docking protocol was applied^58^^,^^59^.

**Figure 4.**
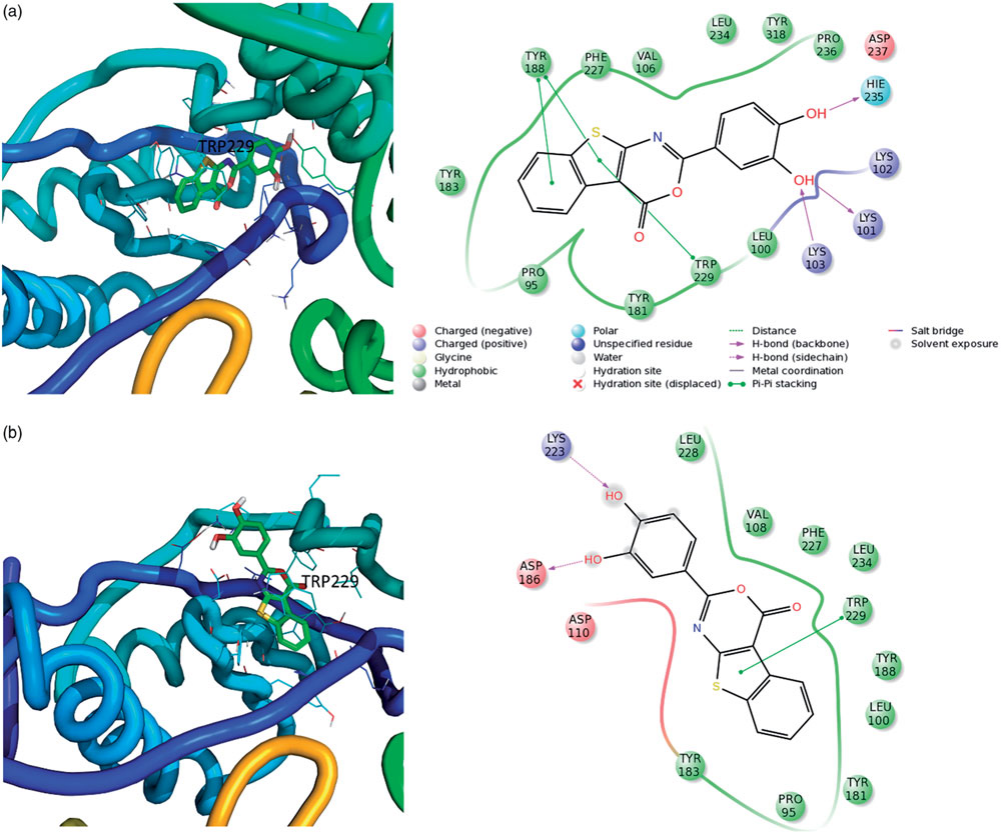
Three-dimensional representation of putative binding modes in (a) pose A and (b) pose B of compound **22** into RT obtained by blind docking experiments and relative 2D representation of the complexes stabilising interactions with binding pocket 1.

**Figure 5.**
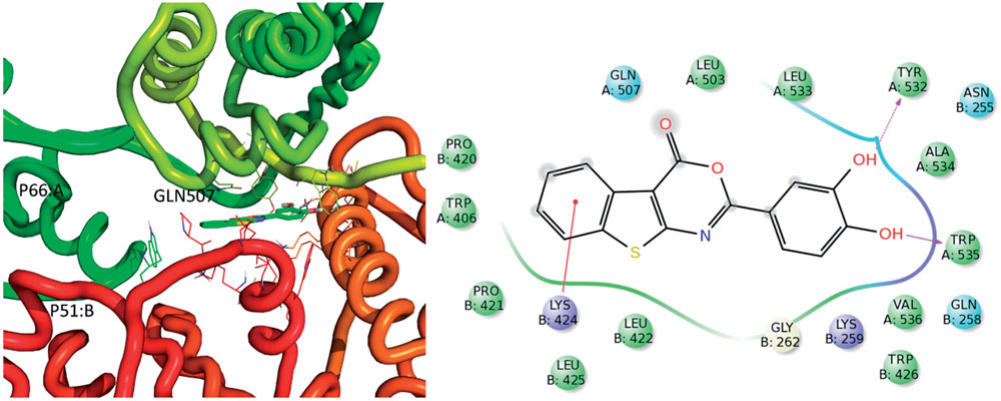
Three-dimensional representation of the putative binding mode obtained by blind docking experiments of compound **22** into RT and relative 2D representation of the complexes stabilising interactions with binding pocket 2.

Due to flexibility of the target and the different shapes of known inhibitors, we decided to carry out ensemble docking experiments using seven different crystal structures^19^. The obtained compound-RT complexes showed that the ligand could bind two different pockets within the RT structure (Figure 4 and Figure 5). The first binding site (pocket 1) is located close to the DNA polymerase catalytic centre partially overlapping the binding pocket of the NNRTIs. The pocket, that possess a “L shape”, could possibly accommodate the compound in two different orientations (Figure 4). According to the docking results, it appears that compound **22** in pose A (Figure 4(a)) could make critical interactions with Y188, W229, H235, K103, and K101. Furthermore, it is stabilised by several hydrophobic interactions with F227, V106, Y318, Y181, and P236. In pose B (Figure 4(b)), compound **22** putative binding mode involves hydrogen bonds with amino acids K223, D186, and W229 and many hydrophobic residues such as L228, V108, F227, L234, Y188, and Y183.

The second putative binding pocket (pocket 2) is in the RNase H domain, between the RNase H active site and the primer grip region, close to the interface of subunits p66 and p51 (Figure 5). Docking experiments suggest that, in this site, the complex is stabilised by a cation-π interaction with K424, and two hydrogen bonds with W535 and Y532. Furthermore, other interactions, mainly with hydrophobic residues of both chains A (p66) and B (p51), contribute to anchoring the compound in this site.

### Site-directed mutagenesis

With the aim of verifying the insights emerged by the computational studies on the binding mode of the compounds, the HIV-1 wt RT structure was modified introducing amino acidic substitutions in the putative binding pocket.

In order to evaluate the impact of the disruption of the main hypothesised interactions for compound **22** in pocket 1, residues Y188 and W229 were substituted with an alanine residue. Moreover, V108 within the site described by Himmel *et al*.^66^ was replaced by a phenylalanine, with the aim to reduce the space at disposal of the compound for the binding in pose B. Analogously, to investigate the effect of mutation occurring in pocket 2, the residue A502 located in the alpha helix close to the putative binding pocket, was substituted with phenylalanine residue, since it was reported that such change causes a movement of alpha helix that reduces the space between the two subunits p51 and p66, thus hindering the entrance of the compound in the pocket^17^.

**Figure 6.**
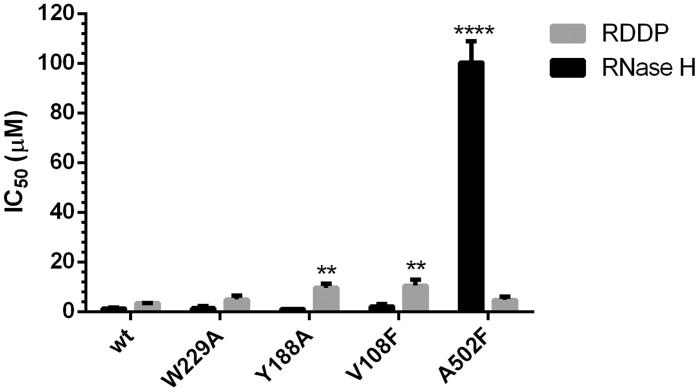
Effects of selected amino acid substitutions in pocket 1 and 2 of HIV-1 RT in the susceptibility to compound **22** in RNase H and RDDP activity assays. *p* values <0.05 (*); *p* values <0.01 (**); *p* values <0.001 (***); *p* values <0.0001(****).

Compound **22** was then tested against both RT-associated enzymatic functions of the mutated RTs, using as comparison the wt enzyme (Figure 6). Regarding the mutations introduced within the pocket 1, results showed that W229A did not significantly affect compound **22** inhibition of neither the RDDP or the RNase H activity. On the other hand, Y188A and V108F substitutions impaired the inhibition of the RDDP (IC_50_=9.64 µM and 10.57 µM, respectively, *p* values=.0039 and .0074, respectively), while no significant effect was observed on the inhibition of the RNase H function. On the contrary, the A502F mutation totally impaired the RNase H inhibition by compound **22** (IC_50_>100 µM, p values<.0001) not significantly affecting the inhibition of the RDDP activity.

**Figure 7.**
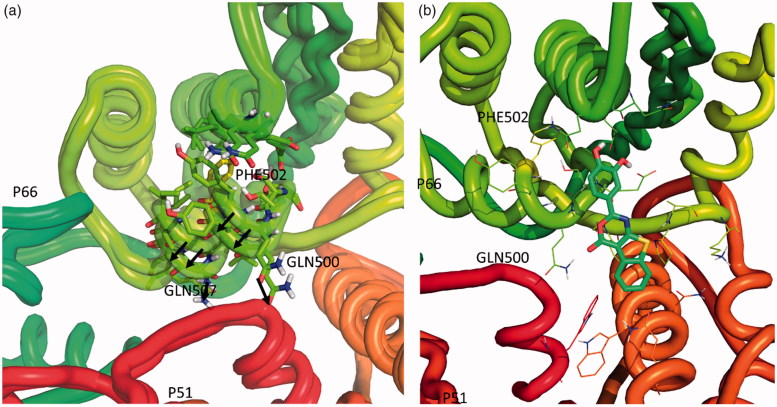
(a) Comparison of wt-RT and A502F-RT; (b) Three-dimensional representation of putative binding mode obtained by docking experiments of compound **22** into A502F-RT.

Overall, these results support the possibility that oxazinone-based compounds bind two different pockets in the RT enzyme, inhibiting the RDDP function binding on pocket 1 and the RNase H function binding on pocket 2. Moreover, the results obtained on the inhibition of the RDDP activity of the RT mutated on pocket 1 suggest the possibility that the binding of compound **22** on this pocket occurs preferentially with the orientation A, partially overlapped with the NNRTIs binding site. Nevertheless, the comparable loss of RDDP activity shown by compound **22** in the presence of V108F and Y188A substitutions (amino acids that belong to the Himmel and NNRTI sites in the L-shaped cavity, respectively) suggests that the compound may assume both the poses, depending on the amino acids substitution. The hypothesised binding in pocket 2 is confirmed by biochemical data on the A502F RT, in which the amino acidic substitution prevents the proper binding within the allosteric pocket as suggested by computational modelling results. Successive docking experiments into the mutated enzyme confirm that, in presence of this mutation, the compound cannot be accommodated in pocket 2 (Figure 7). This can explain the drastic decrease in RNase H inhibitory potency.

### Yonetani–Theorell

**Figure 8.**
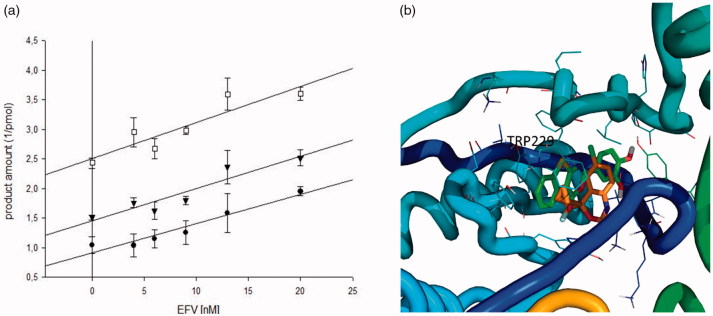
(a) Yonetani-Theorell plot of the combination of EFV and **22** on the HIV-1 RT RNA-dependent DNA polymerase activity of HIV-1 RT wild type. The enzyme was incubated in the presence of EFV alone (●) or in presence of different concentrations of compound **22**: 10 μ (▼) and 30 μM (□); (b) Superimposition of **22** putative binding mode (in green) with experimental EFV (in orange) binding mode reported in pdb 1fk9 ^67^.

In order to confirm the possibility of binding of compound **22** on the NNRTI binding pocket, we performed a Yonetani–Theorell analysis^41^ on the combined effects of compound **22** and EFV on RDDP function. Such an analysis reveals whether the binding of the two inhibitors is or not kinetically mutually exclusive, proving if the simultaneous binding (or inhibition) of two compounds is possible or not. Results showed that the RDDP inhibition of compound **22** and EFV are kinetically mutually exclusive (Figure 8).

### Inhibition of viral replication

Compounds **10** and **20–27** were tested for their ability to inhibit HIV-1 (III_B_ strain) and HIV-2 (ROD strain) replication in acutely infected MT-4 cells, evaluating in parallel their cytotoxicity in the same cells. Unfortunately, none of the compounds affected the viral replication at concentrations lower than those cytotoxic (CC_50_ values ranging from 27 to 300 µM), independently from their ability to inhibit only the RNase H or both the RT functions.

### Liability assays

For the best inhibitors identified in this study, the presence of the catechol moiety emerged as particularly critical in imparting RNase H inhibitory activity. Since catechol has been counted as one among the well-known Pan-Assay Interference Compounds (PAINS) motifs^68^^,^^69^, focused studies have been performed in order to reject the possibility that the inhibitory potency detected in the *in vitro* assays could be related to an artefact. Various mechanisms of assay interference or promiscuous behaviour have been described as responsible for PAINS activity, including metal chelation, compound fluorescence effect, redox activity, cysteine oxidation, and chemical aggregation^68^^,^^70^.

In our study, the intrinsic fluorescence of all the tested compounds have been initially determined to exclude an interference in the readout of the assay used for the determination of the RNase H inhibitory activity. All the compounds emerged no fluorescent at the excitation/emission wavelength (490/528 nm) analysed for the product quantification of the assay. Moreover, as reported above, the magnesium-complexation ability has been investigated for catechol derivatives **22** and **24**. Both the compounds arose unable to chelate Mg^2+^ ions (Figure 2) ruling out both RNase H active site inhibition and an interference with the anti-RNase H activity assay that requires MgCl_2_. Finally, to discard the hypothesis of chemical aggregation, all the compounds were examined by using the ZINC15 remover filter (http://zinc15.docking.org/patterns/home)^71^ and none of them were found as potential aggregator.

## Conclusions

Starting from derivative **1** previously reported by us as an allosteric RNHI, new series of compounds based on cHTC and bicyclic/tricyclic oxazinone scaffolds have been developed in this study. While in the cHTC series the deletion of one of the two aromatic moieties of hit compound **1** was detrimental, oxazinone-based compounds showed an efficient RNase H inhibition when functionalised at the C-2 position with a catechol moiety. Among them, the tricyclic derivatives showed, although to a different extent, the ability to inhibit also the RDDP activity, thus resulting dual-acting compounds. On the other hand, the bicyclic derivatives were completely inactive against RDDP emerging as selective RNHIs. Overall, the most interesting compounds were cycloheptatienooxazinone derivative **10** and benzothienooxazinones **22** and **24**, of which compound **22** resulted in the most potent inhibitor of both RNase H and RDDP functions, with IC_50_ values of 0.53 μM and 2.90 μM, respectively. Spectrophotometric and differential scanning fluorimetry assays showed that the compounds do not chelate the Mg^2+^ nor destabilise the RT conformation, respectively, thus indicating that they are not active site neither RT heterodimer interface inhibitors. Docking studies suggested that they could bind two different pockets within the RT: the first located close to the DNA polymerase catalytic centre partially overlapping the binding pocket of the NNRTIs, and the second in the RNase H domain, between the RNase H active site and the primer grip region, close to the interface of subunits p66 and p51. Site-directed mutagenesis studies confirmed the docking results. Indeed, A502F substitution in pocket 2 completely impaired RNase H inhibition by compound **22**, and a comparable loss of RDDP activity was observed with V108F and Y188A substitutions within the pocket 1 suggesting that the compound may assume two different poses within NNRTI site and that described by Himmel in the L-shaped cavity.

Unfortunately, even exhibiting the ability to inhibit RNase H activity in the sub-micromolar range, the compounds failed in inhibiting the HIV replication in cellular context. This issue and the crucial presence of the catechol moiety to achieve potent anti-RNase H compounds led us to determine their potential as PAINS, which was however ruled-out through appropriate liability assays.

Despite many hypotheses may be postulated on the lack of anti-HIV activity shown by the compounds herein identified, it is worth to remark that this behaviour characterises most of the RNHIs identified so far^10^. Since 1990, when the earliest RNHIs can be traced, several chemotypes have been reported to inhibit HIV RNase H at low micromolar or even nanomolar level, but only a few of them showed significant antiviral activity in cell-based assays^10^. They are mainly metal-chelating active site RNHIs which however inhibited also other essential enzymes within HIV replicative cycle (IN or RT DNA polymerase), thus not permitting to evaluate the exact contribution of the RNase H inhibition to the antiviral activity. Recently, Vernekar *et al.* demonstrated that an ultrapotent RNase H biochemical inhibition (low nanomolar) is required to effectively compete against RNA:DNA duplex substrate while achieving only a moderate level of antiviral activity (low micromolar to sub-micromolar)^72^. For a potent RNase H active-site inhibitor devoid of anti-HIV activity, the authors proposed two different binding modes, in the presence or absence of the substrate, with a loss of a key interaction in the presence of the duplex.

We also observed a weaker anti-RNase H activity when the compounds were added after the RNA:DNA duplex substrate, as measured for compound **10** that was tenfold less active (IC_50_=11 μM *vs* 1.1 μM). However, for our allosteric inhibitors, we cannot properly evaluate the binding mode in the presence/absence of the substrate by docking experiments. Indeed, despite the efforts made in past^73^^,^^74^ and the recent mechanistic studies published^75^, the crystal structures of RT-DNA-RNA hybrid complexes are artefacts obtained with artificial crosslinkers, nicked nucleic acids, ion substitutions and NNRTI addiction to lock the structure in a conformation compatible with RNase H cleavage. In fact, complexes without a NNRTI show the duplex trajectory far from the RNase H catalytic site. At the moment, the most probable hypothesis is that the RT could switch between two structural states, one competent for polymerisation and the other for RNA degradation activity^74^. Certainly, it is known that conformational flexibility of both enzyme and RNA/DNA hybrid does not help structural studies and therefore also computational studies.

Although the inhibitors until now reported clearly highlighted that it is extremely difficult to achieve potent antiviral activity, the RNase H remains a valid target that is worth to be further investigated to achieve for an alternative anti-HIV treatment.
